# Measurements of the production cross section of a $Z$ boson in association with jets in *pp* collisions at $\sqrt{s} = 13$ TeV with the ATLAS detector

**DOI:** 10.1140/epjc/s10052-017-4900-z

**Published:** 2017-05-31

**Authors:** M. Aaboud, G. Aad, B. Abbott, J. Abdallah, O. Abdinov, B. Abeloos, R. Aben, O. S. AbouZeid, N. L. Abraham, H. Abramowicz, H. Abreu, R. Abreu, Y. Abulaiti, B. S. Acharya, S. Adachi, L. Adamczyk, D. L. Adams, J. Adelman, S. Adomeit, T. Adye, A. A. Affolder, T. Agatonovic-Jovin, J. A. Aguilar-Saavedra, S. P. Ahlen, F. Ahmadov, G. Aielli, H. Akerstedt, T. P. A. Åkesson, A. V. Akimov, G. L. Alberghi, J. Albert, S. Albrand, M. J. Alconada Verzini, M. Aleksa, I. N. Aleksandrov, C. Alexa, G. Alexander, T. Alexopoulos, M. Alhroob, B. Ali, M. Aliev, G. Alimonti, J. Alison, S. P. Alkire, B. M. M. Allbrooke, B. W. Allen, P. P. Allport, A. Aloisio, A. Alonso, F. Alonso, C. Alpigiani, A. A. Alshehri, M. Alstaty, B. Alvarez Gonzalez, D. Álvarez Piqueras, M. G. Alviggi, B. T. Amadio, Y. Amaral Coutinho, C. Amelung, D. Amidei, S. P. Amor Dos Santos, A. Amorim, S. Amoroso, G. Amundsen, C. Anastopoulos, L. S. Ancu, N. Andari, T. Andeen, C. F. Anders, G. Anders, J. K. Anders, K. J. Anderson, A. Andreazza, V. Andrei, S. Angelidakis, I. Angelozzi, A. Angerami, F. Anghinolfi, A. V. Anisenkov, N. Anjos, A. Annovi, C. Antel, M. Antonelli, A. Antonov, D. J. Antrim, F. Anulli, M. Aoki, L. Aperio Bella, G. Arabidze, Y. Arai, J. P. Araque, A. T. H. Arce, F. A. Arduh, J-F. Arguin, S. Argyropoulos, M. Arik, A. J. Armbruster, L. J. Armitage, O. Arnaez, H. Arnold, M. Arratia, O. Arslan, A. Artamonov, G. Artoni, S. Artz, S. Asai, N. Asbah, A. Ashkenazi, B. Åsman, L. Asquith, K. Assamagan, R. Astalos, M. Atkinson, N. B. Atlay, K. Augsten, G. Avolio, B. Axen, M. K. Ayoub, G. Azuelos, M. A. Baak, A. E. Baas, M. J. Baca, H. Bachacou, K. Bachas, M. Backes, M. Backhaus, P. Bagiacchi, P. Bagnaia, Y. Bai, J. T. Baines, M. Bajic, O. K. Baker, E. M. Baldin, P. Balek, T. Balestri, F. Balli, W. K. Balunas, E. Banas, Sw. Banerjee, A. A. E. Bannoura, L. Barak, E. L. Barberio, D. Barberis, M. Barbero, T. Barillari, M-S Barisits, T. Barklow, N. Barlow, S. L. Barnes, B. M. Barnett, R. M. Barnett, Z. Barnovska-Blenessy, A. Baroncelli, G. Barone, A. J. Barr, L. Barranco Navarro, F. Barreiro, J. Barreiro Guimarães da Costa, R. Bartoldus, A. E. Barton, P. Bartos, A. Basalaev, A. Bassalat, R. L. Bates, S. J. Batista, J. R. Batley, M. Battaglia, M. Bauce, F. Bauer, H. S. Bawa, J. B. Beacham, M. D. Beattie, T. Beau, P. H. Beauchemin, P. Bechtle, H. P. Beck, K. Becker, M. Becker, M. Beckingham, C. Becot, A. J. Beddall, A. Beddall, V. A. Bednyakov, M. Bedognetti, C. P. Bee, L. J. Beemster, T. A. Beermann, M. Begel, J. K. Behr, A. S. Bell, G. Bella, L. Bellagamba, A. Bellerive, M. Bellomo, K. Belotskiy, O. Beltramello, N. L. Belyaev, O. Benary, D. Benchekroun, M. Bender, K. Bendtz, N. Benekos, Y. Benhammou, E. Benhar Noccioli, J. Benitez, D. P. Benjamin, J. R. Bensinger, S. Bentvelsen, L. Beresford, M. Beretta, D. Berge, E. Bergeaas Kuutmann, N. Berger, J. Beringer, S. Berlendis, N. R. Bernard, C. Bernius, F. U. Bernlochner, T. Berry, P. Berta, C. Bertella, G. Bertoli, F. Bertolucci, I. A. Bertram, C. Bertsche, D. Bertsche, G. J. Besjes, O. Bessidskaia Bylund, M. Bessner, N. Besson, C. Betancourt, A. Bethani, S. Bethke, A. J. Bevan, R. M. Bianchi, M. Bianco, O. Biebel, D. Biedermann, R. Bielski, N. V. Biesuz, M. Biglietti, J. Bilbao De Mendizabal, T. R. V. Billoud, H. Bilokon, M. Bindi, A. Bingul, C. Bini, S. Biondi, T. Bisanz, D. M. Bjergaard, C. W. Black, J. E. Black, K. M. Black, D. Blackburn, R. E. Blair, T. Blazek, I. Bloch, C. Blocker, A. Blue, W. Blum, U. Blumenschein, S. Blunier, G. J. Bobbink, V. S. Bobrovnikov, S. S. Bocchetta, A. Bocci, C. Bock, M. Boehler, D. Boerner, J. A. Bogaerts, D. Bogavac, A. G. Bogdanchikov, C. Bohm, V. Boisvert, P. Bokan, T. Bold, A. S. Boldyrev, M. Bomben, M. Bona, M. Boonekamp, A. Borisov, G. Borissov, J. Bortfeldt, D. Bortoletto, V. Bortolotto, K. Bos, D. Boscherini, M. Bosman, J. D. Bossio Sola, J. Boudreau, J. Bouffard, E. V. Bouhova-Thacker, D. Boumediene, C. Bourdarios, S. K. Boutle, A. Boveia, J. Boyd, I. R. Boyko, J. Bracinik, A. Brandt, G. Brandt, O. Brandt, U. Bratzler, B. Brau, J. E. Brau, W. D. Breaden Madden, K. Brendlinger, A. J. Brennan, L. Brenner, R. Brenner, S. Bressler, T. M. Bristow, D. Britton, D. Britzger, F. M. Brochu, I. Brock, R. Brock, G. Brooijmans, T. Brooks, W. K. Brooks, J. Brosamer, E. Brost, J. H Broughton, P. A. Bruckman de Renstrom, D. Bruncko, R. Bruneliere, A. Bruni, G. Bruni, L. S. Bruni, BH Brunt, M. Bruschi, N. Bruscino, P. Bryant, L. Bryngemark, T. Buanes, Q. Buat, P. Buchholz, A. G. Buckley, I. A. Budagov, F. Buehrer, M. K. Bugge, O. Bulekov, D. Bullock, H. Burckhart, S. Burdin, C. D. Burgard, A. M. Burger, B. Burghgrave, K. Burka, S. Burke, I. Burmeister, J. T. P. Burr, E. Busato, D. Büscher, V. Büscher, P. Bussey, J. M. Butler, C. M. Buttar, J. M. Butterworth, P. Butti, W. Buttinger, A. Buzatu, A. R. Buzykaev, S. Cabrera Urbán, D. Caforio, V. M. Cairo, O. Cakir, N. Calace, P. Calafiura, A. Calandri, G. Calderini, P. Calfayan, G. Callea, L. P. Caloba, S. Calvente Lopez, D. Calvet, S. Calvet, T. P. Calvet, R. Camacho Toro, S. Camarda, P. Camarri, D. Cameron, R. Caminal Armadans, C. Camincher, S. Campana, M. Campanelli, A. Camplani, A. Campoverde, V. Canale, A. Canepa, M. Cano Bret, J. Cantero, T. Cao, M. D. M. Capeans Garrido, I. Caprini, M. Caprini, M. Capua, R. M. Carbone, R. Cardarelli, F. Cardillo, I. Carli, T. Carli, G. Carlino, L. Carminati, R. M. D. Carney, S. Caron, E. Carquin, G. D. Carrillo-Montoya, J. R. Carter, J. Carvalho, D. Casadei, M. P. Casado, M. Casolino, D. W. Casper, E. Castaneda-Miranda, R. Castelijn, A. Castelli, V. Castillo Gimenez, N. F. Castro, A. Catinaccio, J. R. Catmore, A. Cattai, J. Caudron, V. Cavaliere, E. Cavallaro, D. Cavalli, M. Cavalli-Sforza, V. Cavasinni, F. Ceradini, L. Cerda Alberich, A. S. Cerqueira, A. Cerri, L. Cerrito, F. Cerutti, A. Cervelli, S. A. Cetin, A. Chafaq, D. Chakraborty, S. K. Chan, Y. L. Chan, P. Chang, J. D. Chapman, D. G. Charlton, A. Chatterjee, C. C. Chau, C. A. Chavez Barajas, S. Che, S. Cheatham, A. Chegwidden, S. Chekanov, S. V. Chekulaev, G. A. Chelkov, M. A. Chelstowska, C. Chen, H. Chen, K. Chen, S. Chen, S. Chen, X. Chen, Y. Chen, H. C. Cheng, H. J. Cheng, Y. Cheng, A. Cheplakov, E. Cheremushkina, R. Cherkaoui El Moursli, V. Chernyatin, E. Cheu, L. Chevalier, V. Chiarella, G. Chiarelli, G. Chiodini, A. S. Chisholm, A. Chitan, M. V. Chizhov, K. Choi, A. R. Chomont, S. Chouridou, B. K. B. Chow, V. Christodoulou, D. Chromek-Burckhart, J. Chudoba, A. J. Chuinard, J. J. Chwastowski, L. Chytka, G. Ciapetti, A. K. Ciftci, D. Cinca, V. Cindro, I. A. Cioara, C. Ciocca, A. Ciocio, F. Cirotto, Z. H. Citron, M. Citterio, M. Ciubancan, A. Clark, B. L. Clark, M. R. Clark, P. J. Clark, R. N. Clarke, C. Clement, Y. Coadou, M. Cobal, A. Coccaro, J. Cochran, L. Colasurdo, B. Cole, A. P. Colijn, J. Collot, T. Colombo, G. Compostella, P. Conde Muiño, E. Coniavitis, S. H. Connell, I. A. Connelly, V. Consorti, S. Constantinescu, G. Conti, F. Conventi, M. Cooke, B. D. Cooper, A. M. Cooper-Sarkar, F. Cormier, K. J. R. Cormier, T. Cornelissen, M. Corradi, F. Corriveau, A. Cortes-Gonzalez, G. Cortiana, G. Costa, M. J. Costa, D. Costanzo, G. Cottin, G. Cowan, B. E. Cox, K. Cranmer, S. J. Crawley, G. Cree, S. Crépé-Renaudin, F. Crescioli, W. A. Cribbs, M. Crispin Ortuzar, M. Cristinziani, V. Croft, G. Crosetti, A. Cueto, T. Cuhadar Donszelmann, J. Cummings, M. Curatolo, J. Cúth, H. Czirr, P. Czodrowski, G. D’amen, S. D’Auria, M. D’Onofrio, M. J. Da Cunha Sargedas De Sousa, C. Da Via, W. Dabrowski, T. Dado, T. Dai, O. Dale, F. Dallaire, C. Dallapiccola, M. Dam, J. R. Dandoy, N. P. Dang, A. C. Daniells, N. S. Dann, M. Danninger, M. Dano Hoffmann, V. Dao, G. Darbo, S. Darmora, J. Dassoulas, A. Dattagupta, W. Davey, C. David, T. Davidek, M. Davies, P. Davison, E. Dawe, I. Dawson, K. De, R. de Asmundis, A. De Benedetti, S. De Castro, S. De Cecco, N. De Groot, P. de Jong, H. De la Torre, F. De Lorenzi, A. De Maria, D. De Pedis, A. De Salvo, U. De Sanctis, A. De Santo, J. B. De Vivie De Regie, W. J. Dearnaley, R. Debbe, C. Debenedetti, D. V. Dedovich, N. Dehghanian, I. Deigaard, M. Del Gaudio, J. Del Peso, T. Del Prete, D. Delgove, F. Deliot, C. M. Delitzsch, A. Dell’Acqua, L. Dell’Asta, M. Dell’Orso, M. Della Pietra, D. della Volpe, M. Delmastro, P. A. Delsart, D. A. DeMarco, S. Demers, M. Demichev, A. Demilly, S. P. Denisov, D. Denysiuk, D. Derendarz, J. E. Derkaoui, F. Derue, P. Dervan, K. Desch, C. Deterre, K. Dette, P. O. Deviveiros, A. Dewhurst, S. Dhaliwal, A. Di Ciaccio, L. Di Ciaccio, W. K. Di Clemente, C. Di Donato, A. Di Girolamo, B. Di Girolamo, B. Di Micco, R. Di Nardo, K. F. Di Petrillo, A. Di Simone, R. Di Sipio, D. Di Valentino, C. Diaconu, M. Diamond, F. A. Dias, M. A. Diaz, E. B. Diehl, J. Dietrich, S. Díez Cornell, A. Dimitrievska, J. Dingfelder, P. Dita, S. Dita, F. Dittus, F. Djama, T. Djobava, J. I. Djuvsland, M. A. B. do Vale, D. Dobos, M. Dobre, C. Doglioni, J. Dolejsi, Z. Dolezal, M. Donadelli, S. Donati, P. Dondero, J. Donini, J. Dopke, A. Doria, M. T. Dova, A. T. Doyle, E. Drechsler, M. Dris, Y. Du, J. Duarte-Campderros, E. Duchovni, G. Duckeck, O. A. Ducu, D. Duda, A. Dudarev, A. Chr. Dudder, E. M. Duffield, L. Duflot, M. Dührssen, M. Dumancic, A. K. Duncan, M. Dunford, H. Duran Yildiz, M. Düren, A. Durglishvili, D. Duschinger, B. Dutta, M. Dyndal, C. Eckardt, K. M. Ecker, R. C. Edgar, N. C. Edwards, T. Eifert, G. Eigen, K. Einsweiler, T. Ekelof, M. El Kacimi, V. Ellajosyula, M. Ellert, S. Elles, F. Ellinghaus, A. A. Elliot, N. Ellis, J. Elmsheuser, M. Elsing, D. Emeliyanov, Y. Enari, O. C. Endner, J. S. Ennis, J. Erdmann, A. Ereditato, G. Ernis, J. Ernst, M. Ernst, S. Errede, E. Ertel, M. Escalier, H. Esch, C. Escobar, B. Esposito, A. I. Etienvre, E. Etzion, H. Evans, A. Ezhilov, F. Fabbri, L. Fabbri, G. Facini, R. M. Fakhrutdinov, S. Falciano, R. J. Falla, J. Faltova, Y. Fang, M. Fanti, A. Farbin, A. Farilla, C. Farina, E. M. Farina, T. Farooque, S. Farrell, S. M. Farrington, P. Farthouat, F. Fassi, P. Fassnacht, D. Fassouliotis, M. Faucci Giannelli, A. Favareto, W. J. Fawcett, L. Fayard, O. L. Fedin, W. Fedorko, S. Feigl, L. Feligioni, C. Feng, E. J. Feng, H. Feng, A. B. Fenyuk, L. Feremenga, P. Fernandez Martinez, S. Fernandez Perez, J. Ferrando, A. Ferrari, P. Ferrari, R. Ferrari, D. E. Ferreira de Lima, A. Ferrer, D. Ferrere, C. Ferretti, F. Fiedler, A. Filipčič, M. Filipuzzi, F. Filthaut, M. Fincke-Keeler, K. D. Finelli, M. C. N. Fiolhais, L. Fiorini, A. Fischer, C. Fischer, J. Fischer, W. C. Fisher, N. Flaschel, I. Fleck, P. Fleischmann, G. T. Fletcher, R. R. M. Fletcher, T. Flick, B. M. Flierl, L. R. Flores Castillo, M. J. Flowerdew, G. T. Forcolin, A. Formica, A. Forti, A. G. Foster, D. Fournier, H. Fox, S. Fracchia, P. Francavilla, M. Franchini, D. Francis, L. Franconi, M. Franklin, M. Frate, M. Fraternali, D. Freeborn, S. M. Fressard-Batraneanu, F. Friedrich, D. Froidevaux, J. A. Frost, C. Fukunaga, E. Fullana Torregrosa, T. Fusayasu, J. Fuster, C. Gabaldon, O. Gabizon, A. Gabrielli, A. Gabrielli, G. P. Gach, S. Gadatsch, G. Gagliardi, L. G. Gagnon, P. Gagnon, C. Galea, B. Galhardo, E. J. Gallas, B. J. Gallop, P. Gallus, G. Galster, K. K. Gan, S. Ganguly, J. Gao, Y. Gao, Y. S. Gao, F. M. Garay Walls, C. García, J. E. García Navarro, M. Garcia-Sciveres, R. W. Gardner, N. Garelli, V. Garonne, A. Gascon Bravo, K. Gasnikova, C. Gatti, A. Gaudiello, G. Gaudio, L. Gauthier, I. L. Gavrilenko, C. Gay, G. Gaycken, E. N. Gazis, Z. Gecse, C. N. P. Gee, Ch. Geich-Gimbel, M. Geisen, M. P. Geisler, K. Gellerstedt, C. Gemme, M. H. Genest, C. Geng, S. Gentile, C. Gentsos, S. George, D. Gerbaudo, A. Gershon, S. Ghasemi, M. Ghneimat, B. Giacobbe, S. Giagu, P. Giannetti, S. M. Gibson, M. Gignac, M. Gilchriese, T. P. S. Gillam, D. Gillberg, G. Gilles, D. M. Gingrich, N. Giokaris, M. P. Giordani, F. M. Giorgi, P. F. Giraud, P. Giromini, D. Giugni, F. Giuli, C. Giuliani, M. Giulini, B. K. Gjelsten, S. Gkaitatzis, I. Gkialas, E. L. Gkougkousis, L. K. Gladilin, C. Glasman, J. Glatzer, P. C. F. Glaysher, A. Glazov, M. Goblirsch-Kolb, J. Godlewski, S. Goldfarb, T. Golling, D. Golubkov, A. Gomes, R. Gonçalo, J. Goncalves Pinto Firmino Da Costa, G. Gonella, L. Gonella, A. Gongadze, S. González de la Hoz, S. Gonzalez-Sevilla, L. Goossens, P. A. Gorbounov, H. A. Gordon, I. Gorelov, B. Gorini, E. Gorini, A. Gorišek, E. Gornicki, A. T. Goshaw, C. Gössling, M. I. Gostkin, C. R. Goudet, D. Goujdami, A. G. Goussiou, N. Govender, E. Gozani, L. Graber, I. Grabowska-Bold, P. O. J. Gradin, P. Grafström, J. Gramling, E. Gramstad, S. Grancagnolo, V. Gratchev, P. M. Gravila, H. M. Gray, E. Graziani, Z. D. Greenwood, C. Grefe, K. Gregersen, I. M. Gregor, P. Grenier, K. Grevtsov, J. Griffiths, A. A. Grillo, K. Grimm, S. Grinstein, Ph. Gris, J.-F. Grivaz, S. Groh, E. Gross, J. Grosse-Knetter, G. C. Grossi, Z. J. Grout, L. Guan, W. Guan, J. Guenther, F. Guescini, D. Guest, O. Gueta, B. Gui, E. Guido, T. Guillemin, S. Guindon, U. Gul, C. Gumpert, J. Guo, Y. Guo, R. Gupta, S. Gupta, G. Gustavino, P. Gutierrez, N. G. Gutierrez Ortiz, C. Gutschow, C. Guyot, C. Gwenlan, C. B. Gwilliam, A. Haas, C. Haber, H. K. Hadavand, A. Hadef, S. Hageböck, M. Hagihara, Z. Hajduk, H. Hakobyan, M. Haleem, J. Haley, G. Halladjian, G. D. Hallewell, K. Hamacher, P. Hamal, K. Hamano, A. Hamilton, G. N. Hamity, P. G. Hamnett, L. Han, K. Hanagaki, K. Hanawa, M. Hance, B. Haney, P. Hanke, R. Hanna, J. B. Hansen, J. D. Hansen, M. C. Hansen, P. H. Hansen, K. Hara, A. S. Hard, T. Harenberg, F. Hariri, S. Harkusha, R. D. Harrington, P. F. Harrison, F. Hartjes, N. M. Hartmann, M. Hasegawa, Y. Hasegawa, A. Hasib, S. Hassani, S. Haug, R. Hauser, L. Hauswald, M. Havranek, C. M. Hawkes, R. J. Hawkings, D. Hayakawa, D. Hayden, C. P. Hays, J. M. Hays, H. S. Hayward, S. J. Haywood, S. J. Head, T. Heck, V. Hedberg, L. Heelan, S. Heim, T. Heim, B. Heinemann, J. J. Heinrich, L. Heinrich, C. Heinz, J. Hejbal, L. Helary, S. Hellman, C. Helsens, J. Henderson, R. C. W. Henderson, Y. Heng, S. Henkelmann, A. M. Henriques Correia, S. Henrot-Versille, G. H. Herbert, H. Herde, V. Herget, Y. Hernández Jiménez, G. Herten, R. Hertenberger, L. Hervas, G. G. Hesketh, N. P. Hessey, J. W. Hetherly, E. Higón-Rodriguez, E. Hill, J. C. Hill, K. H. Hiller, S. J. Hillier, I. Hinchliffe, E. Hines, R. R. Hinman, M. Hirose, D. Hirschbuehl, X. Hoad, J. Hobbs, N. Hod, M. C. Hodgkinson, P. Hodgson, A. Hoecker, M. R. Hoeferkamp, F. Hoenig, D. Hohn, T. R. Holmes, M. Homann, T. Honda, T. M. Hong, B. H. Hooberman, W. H. Hopkins, Y. Horii, A. J. Horton, J-Y. Hostachy, S. Hou, A. Hoummada, J. Howarth, J. Hoya, M. Hrabovsky, I. Hristova, J. Hrivnac, T. Hryn’ova, A. Hrynevich, P. J. Hsu, S.-C. Hsu, Q. Hu, S. Hu, Y. Huang, Z. Hubacek, F. Hubaut, F. Huegging, T. B. Huffman, E. W. Hughes, G. Hughes, M. Huhtinen, P. Huo, N. Huseynov, J. Huston, J. Huth, G. Iacobucci, G. Iakovidis, I. Ibragimov, L. Iconomidou-Fayard, E. Ideal, P. Iengo, O. Igonkina, T. Iizawa, Y. Ikegami, M. Ikeno, Y. Ilchenko, D. Iliadis, N. Ilic, G. Introzzi, P. Ioannou, M. Iodice, K. Iordanidou, V. Ippolito, N. Ishijima, M. Ishino, M. Ishitsuka, R. Ishmukhametov, C. Issever, S. Istin, F. Ito, J. M. Iturbe Ponce, R. Iuppa, W. Iwanski, H. Iwasaki, J. M. Izen, V. Izzo, S. Jabbar, B. Jackson, P. Jackson, V. Jain, K. B. Jakobi, K. Jakobs, S. Jakobsen, T. Jakoubek, D. O. Jamin, D. K. Jana, R. Jansky, J. Janssen, M. Janus, P. A. Janus, G. Jarlskog, N. Javadov, T. Javůrek, M. Javurkova, F. Jeanneau, L. Jeanty, J. Jejelava, G.-Y. Jeng, D. Jennens, P. Jenni, C. Jeske, S. Jézéquel, H. Ji, J. Jia, H. Jiang, Y. Jiang, Z. Jiang, S. Jiggins, J. Jimenez Pena, S. Jin, A. Jinaru, O. Jinnouchi, H. Jivan, P. Johansson, K. A. Johns, W. J. Johnson, K. Jon-And, G. Jones, R. W. L. Jones, S. Jones, T. J. Jones, J. Jongmanns, P. M. Jorge, J. Jovicevic, X. Ju, A. Juste Rozas, M. K. Köhler, A. Kaczmarska, M. Kado, H. Kagan, M. Kagan, S. J. Kahn, T. Kaji, E. Kajomovitz, C. W. Kalderon, A. Kaluza, S. Kama, A. Kamenshchikov, N. Kanaya, S. Kaneti, L. Kanjir, V. A. Kantserov, J. Kanzaki, B. Kaplan, L. S. Kaplan, A. Kapliy, D. Kar, K. Karakostas, A. Karamaoun, N. Karastathis, M. J. Kareem, E. Karentzos, M. Karnevskiy, S. N. Karpov, Z. M. Karpova, K. Karthik, V. Kartvelishvili, A. N. Karyukhin, K. Kasahara, L. Kashif, R. D. Kass, A. Kastanas, Y. Kataoka, C. Kato, A. Katre, J. Katzy, K. Kawade, K. Kawagoe, T. Kawamoto, G. Kawamura, V. F. Kazanin, R. Keeler, R. Kehoe, J. S. Keller, J. J. Kempster, H. Keoshkerian, O. Kepka, B. P. Kerševan, S. Kersten, R. A. Keyes, M. Khader, F. Khalil-zada, A. Khanov, A. G. Kharlamov, T. Kharlamova, T. J. Khoo, V. Khovanskiy, E. Khramov, J. Khubua, S. Kido, C. R. Kilby, H. Y. Kim, S. H. Kim, Y. K. Kim, N. Kimura, O. M. Kind, B. T. King, M. King, J. Kirk, A. E. Kiryunin, T. Kishimoto, D. Kisielewska, F. Kiss, K. Kiuchi, O. Kivernyk, E. Kladiva, M. H. Klein, M. Klein, U. Klein, K. Kleinknecht, P. Klimek, A. Klimentov, R. Klingenberg, T. Klioutchnikova, E.-E. Kluge, P. Kluit, S. Kluth, J. Knapik, E. Kneringer, E. B. F. G. Knoops, A. Knue, A. Kobayashi, D. Kobayashi, T. Kobayashi, M. Kobel, M. Kocian, P. Kodys, T. Koffas, E. Koffeman, N. M. Köhler, T. Koi, H. Kolanoski, M. Kolb, I. Koletsou, A. A. Komar, Y. Komori, T. Kondo, N. Kondrashova, K. Köneke, A. C. König, T. Kono, R. Konoplich, N. Konstantinidis, R. Kopeliansky, S. Koperny, L. Köpke, A. K. Kopp, K. Korcyl, K. Kordas, A. Korn, A. A. Korol, I. Korolkov, E. V. Korolkova, O. Kortner, S. Kortner, T. Kosek, V. V. Kostyukhin, A. Kotwal, A. Koulouris, A. Kourkoumeli-Charalampidi, C. Kourkoumelis, V. Kouskoura, A. B. Kowalewska, R. Kowalewski, T. Z. Kowalski, C. Kozakai, W. Kozanecki, A. S. Kozhin, V. A. Kramarenko, G. Kramberger, D. Krasnopevtsev, M. W. Krasny, A. Krasznahorkay, A. Kravchenko, M. Kretz, J. Kretzschmar, K. Kreutzfeldt, P. Krieger, K. Krizka, K. Kroeninger, H. Kroha, J. Kroll, J. Kroseberg, J. Krstic, U. Kruchonak, H. Krüger, N. Krumnack, M. C. Kruse, M. Kruskal, T. Kubota, H. Kucuk, S. Kuday, J. T. Kuechler, S. Kuehn, A. Kugel, F. Kuger, T. Kuhl, V. Kukhtin, R. Kukla, Y. Kulchitsky, S. Kuleshov, M. Kuna, T. Kunigo, A. Kupco, H. Kurashige, L. L. Kurchaninov, Y. A. Kurochkin, M. G. Kurth, V. Kus, E. S. Kuwertz, M. Kuze, J. Kvita, T. Kwan, D. Kyriazopoulos, A. La Rosa, J. L. La Rosa Navarro, L. La Rotonda, C. Lacasta, F. Lacava, J. Lacey, H. Lacker, D. Lacour, V. R. Lacuesta, E. Ladygin, R. Lafaye, B. Laforge, T. Lagouri, S. Lai, S. Lammers, W. Lampl, E. Lançon, U. Landgraf, M. P. J. Landon, M. C. Lanfermann, V. S. Lang, J. C. Lange, A. J. Lankford, F. Lanni, K. Lantzsch, A. Lanza, S. Laplace, C. Lapoire, J. F. Laporte, T. Lari, F. Lasagni Manghi, M. Lassnig, P. Laurelli, W. Lavrijsen, A. T. Law, P. Laycock, T. Lazovich, M. Lazzaroni, B. Le, O. Le Dortz, E. Le Guirriec, E. P. Le Quilleuc, M. LeBlanc, T. LeCompte, F. Ledroit-Guillon, C. A. Lee, S. C. Lee, L. Lee, B. Lefebvre, G. Lefebvre, M. Lefebvre, F. Legger, C. Leggett, A. Lehan, G. Lehmann Miotto, X. Lei, W. A. Leight, A. G. Leister, M. A. L. Leite, R. Leitner, D. Lellouch, B. Lemmer, K. J. C. Leney, T. Lenz, B. Lenzi, R. Leone, S. Leone, C. Leonidopoulos, S. Leontsinis, G. Lerner, C. Leroy, A. A. J. Lesage, C. G. Lester, M. Levchenko, J. Levêque, D. Levin, L. J. Levinson, M. Levy, D. Lewis, M. Leyton, B. Li, C. Li, H. Li, L. Li, L. Li, Q. Li, S. Li, X. Li, Y. Li, Z. Liang, B. Liberti, A. Liblong, P. Lichard, K. Lie, J. Liebal, W. Liebig, A. Limosani, S. C. Lin, T. H. Lin, B. E. Lindquist, A. E. Lionti, E. Lipeles, A. Lipniacka, M. Lisovyi, T. M. Liss, A. Lister, A. M. Litke, B. Liu, D. Liu, H. Liu, H. Liu, J. Liu, J. B. Liu, K. Liu, L. Liu, M. Liu, Y. L. Liu, Y. Liu, M. Livan, A. Lleres, J. Llorente Merino, S. L. Lloyd, F. Lo Sterzo, E. M. Lobodzinska, P. Loch, F. K. Loebinger, K. M. Loew, A. Loginov, T. Lohse, K. Lohwasser, M. Lokajicek, B. A. Long, J. D. Long, R. E. Long, L. Longo, K. A. Looper, J. A. Lopez, D. Lopez Mateos, B. Lopez Paredes, I. Lopez Paz, A. Lopez Solis, J. Lorenz, N. Lorenzo Martinez, M. Losada, P. J. Lösel, X. Lou, A. Lounis, J. Love, P. A. Love, H. Lu, N. Lu, H. J. Lubatti, C. Luci, A. Lucotte, C. Luedtke, F. Luehring, W. Lukas, L. Luminari, O. Lundberg, B. Lund-Jensen, P. M. Luzi, D. Lynn, R. Lysak, E. Lytken, V. Lyubushkin, H. Ma, L. L. Ma, Y. Ma, G. Maccarrone, A. Macchiolo, C. M. Macdonald, B. Maček, J. Machado Miguens, D. Madaffari, R. Madar, H. J. Maddocks, W. F. Mader, A. Madsen, J. Maeda, S. Maeland, T. Maeno, A. Maevskiy, E. Magradze, J. Mahlstedt, C. Maiani, C. Maidantchik, A. A. Maier, T. Maier, A. Maio, S. Majewski, Y. Makida, N. Makovec, B. Malaescu, Pa. Malecki, V. P. Maleev, F. Malek, U. Mallik, D. Malon, C. Malone, C. Malone, S. Maltezos, S. Malyukov, J. Mamuzic, G. Mancini, L. Mandelli, I. Mandić, J. Maneira, L. Manhaes de Andrade Filho, J. Manjarres Ramos, A. Mann, A. Manousos, B. Mansoulie, J. D. Mansour, R. Mantifel, M. Mantoani, S. Manzoni, L. Mapelli, G. Marceca, L. March, G. Marchiori, M. Marcisovsky, M. Marjanovic, D. E. Marley, F. Marroquim, S. P. Marsden, Z. Marshall, S. Marti-Garcia, B. Martin, T. A. Martin, V. J. Martin, B. Martin dit Latour, M. Martinez, V. I. Martinez Outschoorn, S. Martin-Haugh, V. S. Martoiu, A. C. Martyniuk, A. Marzin, L. Masetti, T. Mashimo, R. Mashinistov, J. Masik, A. L. Maslennikov, I. Massa, L. Massa, P. Mastrandrea, A. Mastroberardino, T. Masubuchi, P. Mättig, J. Mattmann, J. Maurer, S. J. Maxfield, D. A. Maximov, R. Mazini, I. Maznas, S. M. Mazza, N. C. Mc Fadden, G. Mc Goldrick, S. P. Mc Kee, A. McCarn, R. L. McCarthy, T. G. McCarthy, L. I. McClymont, E. F. McDonald, J. A. Mcfayden, G. Mchedlidze, S. J. McMahon, R. A. McPherson, M. Medinnis, S. Meehan, S. Mehlhase, A. Mehta, K. Meier, C. Meineck, B. Meirose, D. Melini, B. R. Mellado Garcia, M. Melo, F. Meloni, S. B. Menary, L. Meng, X. T. Meng, A. Mengarelli, S. Menke, E. Meoni, S. Mergelmeyer, P. Mermod, L. Merola, C. Meroni, F. S. Merritt, A. Messina, J. Metcalfe, A. S. Mete, C. Meyer, C. Meyer, J-P. Meyer, J. Meyer, H. Meyer Zu Theenhausen, F. Miano, R. P. Middleton, S. Miglioranzi, L. Mijović, G. Mikenberg, M. Mikestikova, M. Mikuž, M. Milesi, A. Milic, D. W. Miller, C. Mills, A. Milov, D. A. Milstead, A. A. Minaenko, Y. Minami, I. A. Minashvili, A. I. Mincer, B. Mindur, M. Mineev, Y. Minegishi, Y. Ming, L. M. Mir, K. P. Mistry, T. Mitani, J. Mitrevski, V. A. Mitsou, A. Miucci, P. S. Miyagawa, A. Mizukami, J. U. Mjörnmark, M. Mlynarikova, T. Moa, K. Mochizuki, P. Mogg, S. Mohapatra, S. Molander, R. Moles-Valls, R. Monden, M. C. Mondragon, K. Mönig, J. Monk, E. Monnier, A. Montalbano, J. Montejo Berlingen, F. Monticelli, S. Monzani, R. W. Moore, N. Morange, D. Moreno, M. Moreno Llácer, P. Morettini, S. Morgenstern, D. Mori, T. Mori, M. Morii, M. Morinaga, V. Morisbak, S. Moritz, A. K. Morley, G. Mornacchi, J. D. Morris, L. Morvaj, P. Moschovakos, M. Mosidze, H. J. Moss, J. Moss, K. Motohashi, R. Mount, E. Mountricha, E. J. W. Moyse, S. Muanza, R. D. Mudd, F. Mueller, J. Mueller, R. S. P. Mueller, T. Mueller, D. Muenstermann, P. Mullen, G. A. Mullier, F. J. Munoz Sanchez, J. A. Murillo Quijada, W. J. Murray, H. Musheghyan, M. Muškinja, A. G. Myagkov, M. Myska, B. P. Nachman, O. Nackenhorst, K. Nagai, R. Nagai, K. Nagano, Y. Nagasaka, K. Nagata, M. Nagel, E. Nagy, A. M. Nairz, Y. Nakahama, K. Nakamura, T. Nakamura, I. Nakano, R. F. Naranjo Garcia, R. Narayan, D. I. Narrias Villar, I. Naryshkin, T. Naumann, G. Navarro, R. Nayyar, H. A. Neal, P. Yu. Nechaeva, T. J. Neep, A. Negri, M. Negrini, S. Nektarijevic, C. Nellist, A. Nelson, S. Nemecek, P. Nemethy, A. A. Nepomuceno, M. Nessi, M. S. Neubauer, M. Neumann, R. M. Neves, P. Nevski, P. R. Newman, D. H. Nguyen, T. Nguyen Manh, R. B. Nickerson, R. Nicolaidou, J. Nielsen, A. Nikiforov, V. Nikolaenko, I. Nikolic-Audit, K. Nikolopoulos, J. K. Nilsen, P. Nilsson, Y. Ninomiya, A. Nisati, R. Nisius, T. Nobe, M. Nomachi, I. Nomidis, T. Nooney, S. Norberg, M. Nordberg, N. Norjoharuddeen, O. Novgorodova, S. Nowak, M. Nozaki, L. Nozka, K. Ntekas, E. Nurse, F. Nuti, F. O’grady, D. C. O’Neil, A. A. O’Rourke, V. O’Shea, F. G. Oakham, H. Oberlack, T. Obermann, J. Ocariz, A. Ochi, I. Ochoa, J. P. Ochoa-Ricoux, S. Oda, S. Odaka, H. Ogren, A. Oh, S. H. Oh, C. C. Ohm, H. Ohman, H. Oide, H. Okawa, Y. Okumura, T. Okuyama, A. Olariu, L. F. Oleiro Seabra, S. A. Olivares Pino, D. Oliveira Damazio, A. Olszewski, J. Olszowska, A. Onofre, K. Onogi, P. U. E. Onyisi, M. J. Oreglia, Y. Oren, D. Orestano, N. Orlando, R. S. Orr, B. Osculati, R. Ospanov, G. Otero y Garzon, H. Otono, M. Ouchrif, F. Ould-Saada, A. Ouraou, K. P. Oussoren, Q. Ouyang, M. Owen, R. E. Owen, V. E. Ozcan, N. Ozturk, K. Pachal, A. Pacheco Pages, L. Pacheco Rodriguez, C. Padilla Aranda, S. Pagan Griso, M. Paganini, F. Paige, P. Pais, K. Pajchel, G. Palacino, S. Palazzo, S. Palestini, M. Palka, D. Pallin, E. St. Panagiotopoulou, I. Panagoulias, C. E. Pandini, J. G. Panduro Vazquez, P. Pani, S. Panitkin, D. Pantea, L. Paolozzi, Th. D. Papadopoulou, K. Papageorgiou, A. Paramonov, D. Paredes Hernandez, A. J. Parker, M. A. Parker, K. A. Parker, F. Parodi, J. A. Parsons, U. Parzefall, V. R. Pascuzzi, E. Pasqualucci, S. Passaggio, Fr. Pastore, G. Pásztor, S. Pataraia, J. R. Pater, T. Pauly, J. Pearce, B. Pearson, L. E. Pedersen, S. Pedraza Lopez, R. Pedro, S. V. Peleganchuk, O. Penc, C. Peng, H. Peng, J. Penwell, B. S. Peralva, M. M. Perego, D. V. Perepelitsa, E. Perez Codina, L. Perini, H. Pernegger, S. Perrella, R. Peschke, V. D. Peshekhonov, K. Peters, R. F. Y. Peters, B. A. Petersen, T. C. Petersen, E. Petit, A. Petridis, C. Petridou, P. Petroff, E. Petrolo, M. Petrov, F. Petrucci, N. E. Pettersson, A. Peyaud, R. Pezoa, P. W. Phillips, G. Piacquadio, E. Pianori, A. Picazio, E. Piccaro, M. Piccinini, M. A. Pickering, R. Piegaia, J. E. Pilcher, A. D. Pilkington, A. W. J. Pin, M. Pinamonti, J. L. Pinfold, A. Pingel, S. Pires, H. Pirumov, M. Pitt, L. Plazak, M.-A. Pleier, V. Pleskot, E. Plotnikova, D. Pluth, R. Poettgen, L. Poggioli, D. Pohl, G. Polesello, A. Poley, A. Policicchio, R. Polifka, A. Polini, C. S. Pollard, V. Polychronakos, K. Pommès, L. Pontecorvo, B. G. Pope, G. A. Popeneciu, A. Poppleton, S. Pospisil, K. Potamianos, I. N. Potrap, C. J. Potter, C. T. Potter, G. Poulard, J. Poveda, V. Pozdnyakov, M. E. Pozo Astigarraga, P. Pralavorio, A. Pranko, S. Prell, D. Price, L. E. Price, M. Primavera, S. Prince, K. Prokofiev, F. Prokoshin, S. Protopopescu, J. Proudfoot, M. Przybycien, D. Puddu, M. Purohit, P. Puzo, J. Qian, G. Qin, Y. Qin, A. Quadt, W. B. Quayle, M. Queitsch-Maitland, D. Quilty, S. Raddum, V. Radeka, V. Radescu, S. K. Radhakrishnan, P. Radloff, P. Rados, F. Ragusa, G. Rahal, J. A. Raine, S. Rajagopalan, M. Rammensee, C. Rangel-Smith, M. G. Ratti, D. M. Rauch, F. Rauscher, S. Rave, T. Ravenscroft, I. Ravinovich, M. Raymond, A. L. Read, N. P. Readioff, M. Reale, D. M. Rebuzzi, A. Redelbach, G. Redlinger, R. Reece, R. G. Reed, K. Reeves, L. Rehnisch, J. Reichert, A. Reiss, C. Rembser, H. Ren, M. Rescigno, S. Resconi, O. L. Rezanova, P. Reznicek, R. Rezvani, R. Richter, S. Richter, E. Richter-Was, O. Ricken, M. Ridel, P. Rieck, C. J. Riegel, J. Rieger, O. Rifki, M. Rijssenbeek, A. Rimoldi, M. Rimoldi, L. Rinaldi, B. Ristić, E. Ritsch, I. Riu, F. Rizatdinova, E. Rizvi, C. Rizzi, S. H. Robertson, A. Robichaud-Veronneau, D. Robinson, J. E. M. Robinson, A. Robson, C. Roda, Y. Rodina, A. Rodriguez Perez, D. Rodriguez Rodriguez, S. Roe, C. S. Rogan, O. Røhne, J. Roloff, A. Romaniouk, M. Romano, S. M. Romano Saez, E. Romero Adam, N. Rompotis, M. Ronzani, L. Roos, E. Ros, S. Rosati, K. Rosbach, P. Rose, N.-A. Rosien, V. Rossetti, E. Rossi, L. P. Rossi, J. H. N. Rosten, R. Rosten, M. Rotaru, I. Roth, J. Rothberg, D. Rousseau, A. Rozanov, Y. Rozen, X. Ruan, F. Rubbo, M. S. Rudolph, F. Rühr, A. Ruiz-Martinez, Z. Rurikova, N. A. Rusakovich, A. Ruschke, H. L. Russell, J. P. Rutherfoord, N. Ruthmann, Y. F. Ryabov, M. Rybar, G. Rybkin, S. Ryu, A. Ryzhov, G. F. Rzehorz, A. F. Saavedra, G. Sabato, S. Sacerdoti, H. F-W. Sadrozinski, R. Sadykov, F. Safai Tehrani, P. Saha, M. Sahinsoy, M. Saimpert, T. Saito, H. Sakamoto, Y. Sakurai, G. Salamanna, A. Salamon, J. E. Salazar Loyola, D. Salek, P. H. Sales De Bruin, D. Salihagic, A. Salnikov, J. Salt, D. Salvatore, F. Salvatore, A. Salvucci, A. Salzburger, D. Sammel, D. Sampsonidis, J. Sánchez, V. Sanchez Martinez, A. Sanchez Pineda, H. Sandaker, R. L. Sandbach, M. Sandhoff, C. Sandoval, D. P. C. Sankey, M. Sannino, A. Sansoni, C. Santoni, R. Santonico, H. Santos, I. Santoyo Castillo, K. Sapp, A. Sapronov, J. G. Saraiva, B. Sarrazin, O. Sasaki, K. Sato, E. Sauvan, G. Savage, P. Savard, N. Savic, C. Sawyer, L. Sawyer, J. Saxon, C. Sbarra, A. Sbrizzi, T. Scanlon, D. A. Scannicchio, M. Scarcella, V. Scarfone, J. Schaarschmidt, P. Schacht, B. M. Schachtner, D. Schaefer, L. Schaefer, R. Schaefer, J. Schaeffer, S. Schaepe, S. Schaetzel, U. Schäfer, A. C. Schaffer, D. Schaile, R. D. Schamberger, V. Scharf, V. A. Schegelsky, D. Scheirich, M. Schernau, C. Schiavi, S. Schier, C. Schillo, M. Schioppa, S. Schlenker, K. R. Schmidt-Sommerfeld, K. Schmieden, C. Schmitt, S. Schmitt, S. Schmitz, B. Schneider, U. Schnoor, L. Schoeffel, A. Schoening, B. D. Schoenrock, E. Schopf, M. Schott, J. F. P. Schouwenberg, J. Schovancova, S. Schramm, M. Schreyer, N. Schuh, A. Schulte, M. J. Schultens, H.-C. Schultz-Coulon, H. Schulz, M. Schumacher, B. A. Schumm, Ph. Schune, A. Schwartzman, T. A. Schwarz, H. Schweiger, Ph. Schwemling, R. Schwienhorst, J. Schwindling, T. Schwindt, G. Sciolla, F. Scuri, F. Scutti, J. Searcy, P. Seema, S. C. Seidel, A. Seiden, F. Seifert, J. M. Seixas, G. Sekhniaidze, K. Sekhon, S. J. Sekula, D. M. Seliverstov, N. Semprini-Cesari, C. Serfon, L. Serin, L. Serkin, M. Sessa, R. Seuster, H. Severini, T. Sfiligoj, F. Sforza, A. Sfyrla, E. Shabalina, N. W. Shaikh, L. Y. Shan, R. Shang, J. T. Shank, M. Shapiro, P. B. Shatalov, K. Shaw, S. M. Shaw, A. Shcherbakova, C. Y. Shehu, P. Sherwood, L. Shi, S. Shimizu, C. O. Shimmin, M. Shimojima, S. Shirabe, M. Shiyakova, A. Shmeleva, D. Shoaleh Saadi, M. J. Shochet, S. Shojaii, D. R. Shope, S. Shrestha, E. Shulga, M. A. Shupe, P. Sicho, A. M. Sickles, P. E. Sidebo, E. Sideras Haddad, O. Sidiropoulou, D. Sidorov, A. Sidoti, F. Siegert, Dj. Sijacki, J. Silva, S. B. Silverstein, V. Simak, Lj. Simic, S. Simion, E. Simioni, B. Simmons, D. Simon, M. Simon, P. Sinervo, N. B. Sinev, M. Sioli, G. Siragusa, S. Yu. Sivoklokov, J. Sjölin, M. B. Skinner, H. P. Skottowe, P. Skubic, M. Slater, T. Slavicek, M. Slawinska, K. Sliwa, R. Slovak, V. Smakhtin, B. H. Smart, L. Smestad, J. Smiesko, S. Yu. Smirnov, Y. Smirnov, L. N. Smirnova, O. Smirnova, J. W. Smith, M. N. K. Smith, R. W. Smith, M. Smizanska, K. Smolek, A. A. Snesarev, I. M. Snyder, S. Snyder, R. Sobie, F. Socher, A. Soffer, D. A. Soh, G. Sokhrannyi, C. A. Solans Sanchez, M. Solar, E. Yu. Soldatov, U. Soldevila, A. A. Solodkov, A. Soloshenko, O. V. Solovyanov, V. Solovyev, P. Sommer, H. Son, H. Y. Song, A. Sood, A. Sopczak, V. Sopko, V. Sorin, D. Sosa, C. L. Sotiropoulou, R. Soualah, A. M. Soukharev, D. South, B. C. Sowden, S. Spagnolo, M. Spalla, M. Spangenberg, F. Spanò, D. Sperlich, F. Spettel, R. Spighi, G. Spigo, L. A. Spiller, M. Spousta, R. D. St. Denis, A. Stabile, R. Stamen, S. Stamm, E. Stanecka, R. W. Stanek, C. Stanescu, M. Stanescu-Bellu, M. M. Stanitzki, S. Stapnes, E. A. Starchenko, G. H. Stark, J. Stark, S. H Stark, P. Staroba, P. Starovoitov, S. Stärz, R. Staszewski, P. Steinberg, B. Stelzer, H. J. Stelzer, O. Stelzer-Chilton, H. Stenzel, G. A. Stewart, J. A. Stillings, M. C. Stockton, M. Stoebe, G. Stoicea, P. Stolte, S. Stonjek, A. R. Stradling, A. Straessner, M. E. Stramaglia, J. Strandberg, S. Strandberg, A. Strandlie, M. Strauss, P. Strizenec, R. Ströhmer, D. M. Strom, R. Stroynowski, A. Strubig, S. A. Stucci, B. Stugu, N. A. Styles, D. Su, J. Su, S. Suchek, Y. Sugaya, M. Suk, V. V. Sulin, S. Sultansoy, T. Sumida, S. Sun, X. Sun, J. E. Sundermann, K. Suruliz, C. J. E. Suster, M. R. Sutton, S. Suzuki, M. Svatos, M. Swiatlowski, S. P. Swift, I. Sykora, T. Sykora, D. Ta, C. Taccini, K. Tackmann, J. Taenzer, A. Taffard, R. Tafirout, N. Taiblum, H. Takai, R. Takashima, T. Takeshita, Y. Takubo, M. Talby, A. A. Talyshev, K. G. Tan, J. Tanaka, M. Tanaka, R. Tanaka, S. Tanaka, R. Tanioka, B. B. Tannenwald, S. Tapia Araya, S. Tapprogge, S. Tarem, G. F. Tartarelli, P. Tas, M. Tasevsky, T. Tashiro, E. Tassi, A. Tavares Delgado, Y. Tayalati, A. C. Taylor, G. N. Taylor, P. T. E. Taylor, W. Taylor, F. A. Teischinger, P. Teixeira-Dias, K. K. Temming, D. Temple, H. Ten Kate, P. K. Teng, J. J. Teoh, F. Tepel, S. Terada, K. Terashi, J. Terron, S. Terzo, M. Testa, R. J. Teuscher, T. Theveneaux-Pelzer, J. P. Thomas, J. Thomas-Wilsker, P. D. Thompson, A. S. Thompson, L. A. Thomsen, E. Thomson, M. J. Tibbetts, R. E. Ticse Torres, V. O. Tikhomirov, Yu. A. Tikhonov, S. Timoshenko, P. Tipton, S. Tisserant, K. Todome, T. Todorov, S. Todorova-Nova, J. Tojo, S. Tokár, K. Tokushuku, E. Tolley, L. Tomlinson, M. Tomoto, L. Tompkins, K. Toms, B. Tong, P. Tornambe, E. Torrence, H. Torres, E. Torró Pastor, J. Toth, F. Touchard, D. R. Tovey, T. Trefzger, A. Tricoli, I. M. Trigger, S. Trincaz-Duvoid, M. F. Tripiana, W. Trischuk, B. Trocmé, A. Trofymov, C. Troncon, M. Trottier-McDonald, M. Trovatelli, L. Truong, M. Trzebinski, A. Trzupek, J. C-L. Tseng, P. V. Tsiareshka, G. Tsipolitis, N. Tsirintanis, S. Tsiskaridze, V. Tsiskaridze, E. G. Tskhadadze, K. M. Tsui, I. I. Tsukerman, V. Tsulaia, S. Tsuno, D. Tsybychev, Y. Tu, A. Tudorache, V. Tudorache, T. T. Tulbure, A. N. Tuna, S. A. Tupputi, S. Turchikhin, D. Turgeman, I. Turk Cakir, R. Turra, P. M. Tuts, G. Ucchielli, I. Ueda, M. Ughetto, F. Ukegawa, G. Unal, A. Undrus, G. Unel, F. C. Ungaro, Y. Unno, C. Unverdorben, J. Urban, P. Urquijo, P. Urrejola, G. Usai, J. Usui, L. Vacavant, V. Vacek, B. Vachon, C. Valderanis, E. Valdes Santurio, N. Valencic, S. Valentinetti, A. Valero, L. Valery, S. Valkar, J. A. Valls Ferrer, W. Van Den Wollenberg, P. C. Van Der Deijl, H. van der Graaf, N. van Eldik, P. van Gemmeren, J. Van Nieuwkoop, I. van Vulpen, M. C. van Woerden, M. Vanadia, W. Vandelli, R. Vanguri, A. Vaniachine, P. Vankov, G. Vardanyan, R. Vari, E. W. Varnes, T. Varol, D. Varouchas, A. Vartapetian, K. E. Varvell, J. G. Vasquez, G. A. Vasquez, F. Vazeille, T. Vazquez Schroeder, J. Veatch, V. Veeraraghavan, L. M. Veloce, F. Veloso, S. Veneziano, A. Ventura, M. Venturi, N. Venturi, A. Venturini, V. Vercesi, M. Verducci, W. Verkerke, J. C. Vermeulen, A. Vest, M. C. Vetterli, O. Viazlo, I. Vichou, T. Vickey, O. E. Vickey Boeriu, G. H. A. Viehhauser, S. Viel, L. Vigani, M. Villa, M. Villaplana Perez, E. Vilucchi, M. G. Vincter, V. B. Vinogradov, C. Vittori, I. Vivarelli, S. Vlachos, M. Vlasak, M. Vogel, P. Vokac, G. Volpi, M. Volpi, H. von der Schmitt, E. von Toerne, V. Vorobel, K. Vorobev, M. Vos, R. Voss, J. H. Vossebeld, N. Vranjes, M. Vranjes Milosavljevic, V. Vrba, M. Vreeswijk, R. Vuillermet, I. Vukotic, P. Wagner, W. Wagner, H. Wahlberg, S. Wahrmund, J. Wakabayashi, J. Walder, R. Walker, W. Walkowiak, V. Wallangen, C. Wang, C. Wang, F. Wang, H. Wang, H. Wang, J. Wang, J. Wang, K. Wang, R. Wang, S. M. Wang, T. Wang, W. Wang, C. Wanotayaroj, A. Warburton, C. P. Ward, D. R. Wardrope, A. Washbrook, P. M. Watkins, A. T. Watson, M. F. Watson, G. Watts, S. Watts, B. M. Waugh, S. Webb, M. S. Weber, S. W. Weber, S. A. Weber, J. S. Webster, A. R. Weidberg, B. Weinert, J. Weingarten, C. Weiser, H. Weits, P. S. Wells, T. Wenaus, T. Wengler, S. Wenig, N. Wermes, M. D. Werner, P. Werner, M. Wessels, J. Wetter, K. Whalen, N. L. Whallon, A. M. Wharton, A. White, M. J. White, R. White, D. Whiteson, F. J. Wickens, W. Wiedenmann, M. Wielers, C. Wiglesworth, L. A. M. Wiik-Fuchs, A. Wildauer, F. Wilk, H. G. Wilkens, H. H. Williams, S. Williams, C. Willis, S. Willocq, J. A. Wilson, I. Wingerter-Seez, F. Winklmeier, O. J. Winston, B. T. Winter, M. Wittgen, T. M. H. Wolf, R. Wolff, M. W. Wolter, H. Wolters, S. D. Worm, B. K. Wosiek, J. Wotschack, M. J. Woudstra, K. W. Wozniak, M. Wu, M. Wu, S. L. Wu, X. Wu, Y. Wu, T. R. Wyatt, B. M. Wynne, S. Xella, Z. Xi, D. Xu, L. Xu, B. Yabsley, S. Yacoob, D. Yamaguchi, Y. Yamaguchi, A. Yamamoto, S. Yamamoto, T. Yamanaka, K. Yamauchi, Y. Yamazaki, Z. Yan, H. Yang, H. Yang, Y. Yang, Z. Yang, W-M. Yao, Y. C. Yap, Y. Yasu, E. Yatsenko, K. H. Yau Wong, J. Ye, S. Ye, I. Yeletskikh, E. Yildirim, K. Yorita, R. Yoshida, K. Yoshihara, C. Young, C. J. S. Young, S. Youssef, D. R. Yu, J. Yu, J. M. Yu, J. Yu, L. Yuan, S. P. Y. Yuen, I. Yusuff, B. Zabinski, G. Zacharis, R. Zaidan, A. M. Zaitsev, N. Zakharchuk, J. Zalieckas, A. Zaman, S. Zambito, L. Zanello, D. Zanzi, C. Zeitnitz, M. Zeman, A. Zemla, J. C. Zeng, Q. Zeng, O. Zenin, T. Ženiš, D. Zerwas, D. Zhang, F. Zhang, G. Zhang, H. Zhang, J. Zhang, L. Zhang, L. Zhang, M. Zhang, R. Zhang, R. Zhang, X. Zhang, Z. Zhang, X. Zhao, Y. Zhao, Z. Zhao, A. Zhemchugov, J. Zhong, B. Zhou, C. Zhou, L. Zhou, L. Zhou, M. Zhou, N. Zhou, C. G. Zhu, H. Zhu, J. Zhu, Y. Zhu, X. Zhuang, K. Zhukov, A. Zibell, D. Zieminska, N. I. Zimine, C. Zimmermann, S. Zimmermann, Z. Zinonos, M. Zinser, M. Ziolkowski, L. Živković, G. Zobernig, A. Zoccoli, M. zur Nedden, L. Zwalinski, ATLAS Collaboration

**Affiliations:** 1 Department of Physics, University of Adelaide, Adelaide, SA Australia; 2 Physics Department, SUNY Albany, Albany, NY USA; 3 Department of Physics, University of Alberta, Edmonton, AB Canada; 4 Department of Physics, Ankara University, Ankara, Turkey; 5 Istanbul Aydin University, Istanbul, Turkey; 6 Division of Physics, TOBB University of Economics and Technology, Ankara, Turkey; 7 LAPP, CNRS/IN2P3 and Université Savoie Mont Blanc, Annecy-le-Vieux, France; 8 High Energy Physics Division, Argonne National Laboratory, Argonne, IL USA; 9 Department of Physics, University of Arizona, Tucson, AZ USA; 10 Department of Physics, The University of Texas at Arlington, Arlington, TX USA; 11 Physics Department, National and Kapodistrian University of Athens, Athens, Greece; 12 Physics Department, National Technical University of Athens, Zografou, Greece; 13 Department of Physics, The University of Texas at Austin, Austin, TX USA; 14 Institute of Physics, Azerbaijan Academy of Sciences, Baku, Azerbaijan; 15 Institut de Física d’Altes Energies (IFAE), The Barcelona Institute of Science and Technology, Barcelona, Spain; 16 Institute of Physics, University of Belgrade, Belgrade, Serbia; 17 Department for Physics and Technology, University of Bergen, Bergen, Norway; 18 Physics Division, Lawrence Berkeley National Laboratory and University of California, Berkeley, CA USA; 19 Department of Physics, Humboldt University, Berlin, Germany; 20 Albert Einstein Center for Fundamental Physics and Laboratory for High Energy Physics, University of Bern, Bern, Switzerland; 21 School of Physics and Astronomy, University of Birmingham, Birmingham, UK; 22 Department of Physics, Bogazici University, Istanbul, Turkey; 23 Department of Physics Engineering, Gaziantep University, Gaziantep, Turkey; 24 Faculty of Engineering and Natural Sciences, Istanbul Bilgi University, Istanbul, Turkey; 25 Faculty of Engineering and Natural Sciences, Bahcesehir University, Istanbul, Turkey; 26 Centro de Investigaciones, Universidad Antonio Narino, Bogota, Colombia; 27 INFN Sezione di Bologna, Bologna, Italy; 28 Dipartimento di Fisica e Astronomia, Università di Bologna, Bologna, Italy; 29 Physikalisches Institut, University of Bonn, Bonn, Germany; 30 Department of Physics, Boston University, Boston, MA USA; 31 Department of Physics, Brandeis University, Waltham, MA USA; 32 Universidade Federal do Rio De Janeiro COPPE/EE/IF, Rio de Janeiro, Brazil; 33 Electrical Circuits Department, Federal University of Juiz de Fora (UFJF), Juiz de Fora, Brazil; 34 Federal University of Sao Joao del Rei (UFSJ), Sao Joao del Rei, Brazil; 35 Instituto de Fisica, Universidade de Sao Paulo, Sao Paulo, Brazil; 36 Physics Department, Brookhaven National Laboratory, Upton, NY USA; 37 Transilvania University of Brasov, Brasov, Romania; 38 Horia Hulubei National Institute of Physics and Nuclear Engineering, Bucharest, Romania; 39 Physics Department, National Institute for Research and Development of Isotopic and Molecular Technologies, Cluj-Napoca, Romania; 40 University Politehnica Bucharest, Bucharest, Romania; 41 West University in Timisoara, Timisoara, Romania; 42 Departamento de Física, Universidad de Buenos Aires, Buenos Aires, Argentina; 43 Cavendish Laboratory, University of Cambridge, Cambridge, UK; 44 Department of Physics, Carleton University, Ottawa, ON Canada; 45 CERN, Geneva, Switzerland; 46 Enrico Fermi Institute, University of Chicago, Chicago, IL USA; 47 Departamento de Física, Pontificia Universidad Católica de Chile, Santiago, Chile; 48 Departamento de Física, Universidad Técnica Federico Santa María, Valparaíso, Chile; 49 Institute of High Energy Physics, Chinese Academy of Sciences, Beijing, China; 50 Department of Physics, Nanjing University, Nanjing, Jiangsu China; 51 Physics Department, Tsinghua University, Beijing, 100084 China; 52 Department of Modern Physics, University of Science and Technology of China, Anhui, China; 53 School of Physics, Shandong University, Shandong, China; 54 Department of Physics and Astronomy, Key Laboratory for Particle Physics, Astrophysics and Cosmology,Ministry of Education,Shanghai Key Laboratory for Particle Physics and Cosmology, Shanghai Jiao Tong University (also at PKU-CHEP), Shanghai, China; 55 Laboratoire de Physique Corpusculaire, Université Clermont Auvergne, Université Blaise Pascal, CNRS/IN2P3, Clermont-Ferrand, France; 56 Nevis Laboratory, Columbia University, Irvington, NY USA; 57 Niels Bohr Institute, University of Copenhagen, Kobenhavn, Denmark; 58 INFN Gruppo Collegato di Cosenza, Laboratori Nazionali di Frascati, Frascati, Italy; 59 Dipartimento di Fisica, Università della Calabria, Rende, Italy; 60 Faculty of Physics and Applied Computer Science, AGH University of Science and Technology, Kraków, Poland; 61 Marian Smoluchowski Institute of Physics, Jagiellonian University, Krakow, Poland; 62 Institute of Nuclear Physics Polish Academy of Sciences, Krakow, Poland; 63 Physics Department, Southern Methodist University, Dallas, TX USA; 64 Physics Department, University of Texas at Dallas, Richardson, TX USA; 65 DESY, Hamburg and Zeuthen, Germany; 66 Lehrstuhl für Experimentelle Physik IV, Technische Universität Dortmund, Dortmund, Germany; 67 Institut für Kern- und Teilchenphysik, Technische Universität Dresden, Dresden, Germany; 68 Department of Physics, Duke University, Durham, NC USA; 69 SUPA-School of Physics and Astronomy, University of Edinburgh, Edinburgh, UK; 70 INFN Laboratori Nazionali di Frascati, Frascati, Italy; 71 Fakultät für Mathematik und Physik, Albert-Ludwigs-Universität, Freiburg, Germany; 72 Departement de Physique Nucleaire et Corpusculaire, Université de Genève, Geneva, Switzerland; 73 INFN Sezione di Genova, Genoa, Italy; 74 Dipartimento di Fisica, Università di Genova, Genova, Italy; 75 E. Andronikashvili Institute of Physics, Iv. Javakhishvili Tbilisi State University, Tbilisi, Georgia; 76 High Energy Physics Institute, Tbilisi State University, Tbilisi, Georgia; 77 II Physikalisches Institut, Justus-Liebig-Universität Giessen, Giessen, Germany; 78 SUPA-School of Physics and Astronomy, University of Glasgow, Glasgow, UK; 79 II Physikalisches Institut, Georg-August-Universität, Göttingen, Germany; 80 Laboratoire de Physique Subatomique et de Cosmologie, Université Grenoble-Alpes, CNRS/IN2P3, Grenoble, France; 81 Laboratory for Particle Physics and Cosmology, Harvard University, Cambridge, MA USA; 82 Kirchhoff-Institut für Physik, Ruprecht-Karls-Universität Heidelberg, Heidelberg, Germany; 83 Physikalisches Institut, Ruprecht-Karls-Universität Heidelberg, Heidelberg, Germany; 84 ZITI Institut für technische Informatik, Ruprecht-Karls-Universität Heidelberg, Mannheim, Germany; 85 Faculty of Applied Information Science, Hiroshima Institute of Technology, Hiroshima, Japan; 86 Department of Physics, The Chinese University of Hong Kong, Shatin, NT Hong Kong; 87 Department of Physics, The University of Hong Kong, Hong Kong, China; 88 Department of Physics and Institute for Advanced Study, The Hong Kong University of Science and Technology, Clear Water Bay, Kowloon, Hong Kong, China; 89 Department of Physics, National Tsing Hua University, Taiwan, Taiwan; 90 Department of Physics, Indiana University, Bloomington, IN USA; 91 Institut für Astro- und Teilchenphysik, Leopold-Franzens-Universität, Innsbruck, Austria; 92 University of Iowa, Iowa city, IA USA; 93 Department of Physics and Astronomy, Iowa State University, Ames, IA USA; 94 Joint Institute for Nuclear Research, JINR Dubna, Dubna, Russia; 95 KEK, High Energy Accelerator Research Organization, Tsukuba, Japan; 96 Graduate School of Science, Kobe University, Kobe, Japan; 97 Faculty of Science, Kyoto University, Kyoto, Japan; 98 Kyoto University of Education, Kyoto, Japan; 99 Department of Physics, Kyushu University, Fukuoka, Japan; 100 Instituto de Física La Plata, Universidad Nacional de La Plata and CONICET, La Plata, Argentina; 101 Physics Department, Lancaster University, Lancaster, UK; 102 INFN Sezione di Lecce, Lecce, Italy; 103 Dipartimento di Matematica e Fisica, Università del Salento, Lecce, Italy; 104 Oliver Lodge Laboratory, University of Liverpool, Liverpool, UK; 105 Department of Experimental Particle Physics, Jožef Stefan Institute and Department of Physics, University of Ljubljana, Ljubljana, Slovenia; 106 School of Physics and Astronomy, Queen Mary University of London, London, UK; 107 Department of Physics, Royal Holloway University of London, Surrey, UK; 108 Department of Physics and Astronomy, University College London, London, UK; 109 Louisiana Tech University, Ruston, LA USA; 110 Laboratoire de Physique Nucléaire et de Hautes Energies, UPMC and Université Paris-Diderot and CNRS/IN2P3, Paris, France; 111 Fysiska institutionen, Lunds universitet, Lund, Sweden; 112 Departamento de Fisica Teorica C-15, Universidad Autonoma de Madrid, Madrid, Spain; 113 Institut für Physik, Universität Mainz, Mainz, Germany; 114 School of Physics and Astronomy, University of Manchester, Manchester, UK; 115 CPPM, Aix-Marseille Université and CNRS/IN2P3, Marseille, France; 116 Department of Physics, University of Massachusetts, Amherst, MA USA; 117 Department of Physics, McGill University, Montreal, QC Canada; 118 School of Physics, University of Melbourne, Melbourne, VIC Australia; 119 Department of Physics, The University of Michigan, Ann Arbor, MI USA; 120 Department of Physics and Astronomy, Michigan State University, East Lansing, MI USA; 121 INFN Sezione di Milano, Milan, Italy; 122 Dipartimento di Fisica, Università di Milano, Milano, Italy; 123 B.I. Stepanov Institute of Physics, National Academy of Sciences of Belarus, Minsk, Republic of Belarus; 124 Research Institute for Nuclear Problems of Byelorussian State University, Minsk, Republic of Belarus; 125 Group of Particle Physics, University of Montreal, Montreal, QC Canada; 126 P.N. Lebedev Physical Institute of the Russian Academy of Sciences, Moscow, Russia; 127 Institute for Theoretical and Experimental Physics (ITEP), Moscow, Russia; 128 National Research Nuclear University MEPhI, Moscow, Russia; 129 D.V. Skobeltsyn Institute of Nuclear Physics, M.V. Lomonosov Moscow State University, Moscow, Russia; 130 Fakultät für Physik, Ludwig-Maximilians-Universität München, München, Germany; 131 Max-Planck-Institut für Physik (Werner-Heisenberg-Institut), München, Germany; 132 Nagasaki Institute of Applied Science, Nagasaki, Japan; 133 Graduate School of Science and Kobayashi-Maskawa Institute, Nagoya University, Nagoya, Japan; 134 INFN Sezione di Napoli, Napoli, Italy; 135 Dipartimento di Fisica, Università di Napoli, Napoli, Italy; 136 Department of Physics and Astronomy, University of New Mexico, Albuquerque, NM USA; 137 Institute for Mathematics, Astrophysics and Particle Physics, Radboud University Nijmegen/Nikhef, Nijmegen, Netherlands; 138 Nikhef National Institute for Subatomic Physics and University of Amsterdam, Amsterdam, Netherlands; 139 Department of Physics, Northern Illinois University, DeKalb, IL USA; 140 Budker Institute of Nuclear Physics, SB RAS, Novosibirsk, Russia; 141 Department of Physics, New York University, New York, NY USA; 142 Ohio State University, Columbus, OH USA; 143 Faculty of Science, Okayama University, Okayama, Japan; 144 Homer L. Dodge Department of Physics and Astronomy, University of Oklahoma, Norman, OK USA; 145 Department of Physics, Oklahoma State University, Stillwater, OK USA; 146 Palacký University, RCPTM, Olomouc, Czech Republic; 147 Center for High Energy Physics, University of Oregon, Eugene, OR USA; 148 LAL, University of Paris-Sud, CNRS/IN2P3, Université Paris-Saclay, Orsay, France; 149 Graduate School of Science, Osaka University, Osaka, Japan; 150 Department of Physics, University of Oslo, Oslo, Norway; 151 Department of Physics, Oxford University, Oxford, UK; 152 INFN Sezione di Pavia, Pavia, Italy; 153 Dipartimento di Fisica, Università di Pavia, Pavia, Italy; 154 Department of Physics, University of Pennsylvania, Philadelphia, PA USA; 155 National Research Centre “Kurchatov Institute” B.P. Konstantinov Petersburg Nuclear Physics Institute, St. Petersburg, Russia; 156 INFN Sezione di Pisa, Pisa, Italy; 157 Dipartimento di Fisica E. Fermi, Università di Pisa, Pisa, Italy; 158 Department of Physics and Astronomy, University of Pittsburgh, Pittsburgh, PA USA; 159 Laboratório de Instrumentação e Física Experimental de Partículas-LIP, Lisboa, Portugal; 160 Faculdade de Ciências, Universidade de Lisboa, Lisboa, Portugal; 161 Department of Physics, University of Coimbra, Coimbra, Portugal; 162 Centro de Física Nuclear da Universidade de Lisboa, Lisboa, Portugal; 163 Departamento de Fisica, Universidade do Minho, Braga, Portugal; 164 Departamento de Fisica Teorica y del Cosmos and CAFPE, Universidad de Granada, Granada, Spain; 165 Dep Fisica and CEFITEC of Faculdade de Ciencias e Tecnologia, Universidade Nova de Lisboa, Caparica, Portugal; 166 Institute of Physics, Academy of Sciences of the Czech Republic, Praha, Czech Republic; 167 Czech Technical University in Prague, Praha, Czech Republic; 168 Charles University, Faculty of Mathematics and Physics, Prague, Czech Republic; 169 State Research Center Institute for High Energy Physics (Protvino), NRC KI, Moscow, Russia; 170 Particle Physics Department, Rutherford Appleton Laboratory, Didcot, UK; 171 INFN Sezione di Roma, Roma, Italy; 172 Dipartimento di Fisica, Sapienza Università di Roma, Roma, Italy; 173 INFN Sezione di Roma Tor Vergata, Roma, Italy; 174 Dipartimento di Fisica, Università di Roma Tor Vergata, Roma, Italy; 175 INFN Sezione di Roma Tre, Roma, Italy; 176 Dipartimento di Matematica e Fisica, Università Roma Tre, Roma, Italy; 177 Faculté des Sciences Ain Chock, Réseau Universitaire de Physique des Hautes Energies-Université Hassan II, Casablanca, Morocco; 178 Centre National de l’Energie des Sciences Techniques Nucleaires, Rabat, Morocco; 179 Faculté des Sciences Semlalia, Université Cadi Ayyad, LPHEA-Marrakech, Marrakech, Morocco; 180 Faculté des Sciences, Université Mohamed Premier and LPTPM, Oujda, Morocco; 181 Faculté des Sciences, Université Mohammed V, Rabat, Morocco; 182 DSM/IRFU (Institut de Recherches sur les Lois Fondamentales de l’Univers), CEA Saclay (Commissariat à l’Energie Atomique et aux Energies Alternatives), Gif-sur-Yvette, France; 183 Santa Cruz Institute for Particle Physics, University of California Santa Cruz, Santa Cruz, CA USA; 184 Department of Physics, University of Washington, Seattle, WA USA; 185 Department of Physics and Astronomy, University of Sheffield, Sheffield, UK; 186 Department of Physics, Shinshu University, Nagano, Japan; 187 Fachbereich Physik, Universität Siegen, Siegen, Germany; 188 Department of Physics, Simon Fraser University, Burnaby, BC Canada; 189 SLAC National Accelerator Laboratory, Stanford, CA USA; 190 Faculty of Mathematics, Physics and Informatics, Comenius University, Bratislava, Slovak Republic; 191 Department of Subnuclear Physics, Institute of Experimental Physics of the Slovak Academy of Sciences, Kosice, Slovak Republic; 192 Department of Physics, University of Cape Town, Cape Town, South Africa; 193 Department of Physics, University of Johannesburg, Johannesburg, South Africa; 194 School of Physics, University of the Witwatersrand, Johannesburg, South Africa; 195 Department of Physics, Stockholm University, Stockholm, Sweden; 196 The Oskar Klein Centre, Stockholm, Sweden; 197 Physics Department, Royal Institute of Technology, Stockholm, Sweden; 198 Departments of Physics and Astronomy and Chemistry, Stony Brook University, Stony Brook, NY USA; 199 Department of Physics and Astronomy, University of Sussex, Brighton, UK; 200 School of Physics, University of Sydney, Sydney, SA Australia; 201 Institute of Physics, Academia Sinica, Taipei, Taiwan; 202 Department of Physics, Technion: Israel Institute of Technology, Haifa, Israel; 203 Raymond and Beverly Sackler School of Physics and Astronomy, Tel Aviv University, Tel Aviv, Israel; 204 Department of Physics, Aristotle University of Thessaloniki, Thessaloníki, Greece; 205 International Center for Elementary Particle Physics and Department of Physics, The University of Tokyo, Tokyo, Japan; 206 Graduate School of Science and Technology, Tokyo Metropolitan University, Tokyo, Japan; 207 Department of Physics, Tokyo Institute of Technology, Tokyo, Japan; 208 Tomsk State University, Tomsk, Russia; 209 Department of Physics, University of Toronto, Toronto, ON Canada; 210 INFN-TIFPA, Trento, Italy; 211 University of Trento, Trento, Italy; 212 TRIUMF, Vancouver, BC Canada; 213 Department of Physics and Astronomy, York University, Toronto, ON Canada; 214 Faculty of Pure and Applied Sciences, and Center for Integrated Research in Fundamental Science and Engineering, University of Tsukuba, Tsukuba, Japan; 215 Department of Physics and Astronomy, Tufts University, Medford, MA USA; 216 Department of Physics and Astronomy, University of California Irvine, Irvine, CA USA; 217 INFN Gruppo Collegato di Udine, Sezione di Trieste, Udine, Italy; 218 ICTP, Trieste, Italy; 219 Dipartimento di Chimica, Fisica e Ambiente, Università di Udine, Udine, Italy; 220 Department of Physics and Astronomy, University of Uppsala, Uppsala, Sweden; 221 Department of Physics, University of Illinois, Urbana, IL USA; 222 Instituto de Fisica Corpuscular (IFIC) and Departamento de Fisica Atomica, Molecular y Nuclear and Departamento de Ingeniería Electrónica and Instituto de Microelectrónica de Barcelona (IMB-CNM), University of Valencia and CSIC, Valencia, Spain; 223 Department of Physics, University of British Columbia, Vancouver, BC Canada; 224 Department of Physics and Astronomy, University of Victoria, Victoria, BC Canada; 225 Department of Physics, University of Warwick, Coventry, UK; 226 Waseda University, Tokyo, Japan; 227 Department of Particle Physics, The Weizmann Institute of Science, Rehovot, Israel; 228 Department of Physics, University of Wisconsin, Madison, WI USA; 229 Fakultät für Physik und Astronomie, Julius-Maximilians-Universität, Würzburg, Germany; 230 Fakultät für Mathematik und Naturwissenschaften, Fachgruppe Physik, Bergische Universität Wuppertal, Wuppertal, Germany; 231 Department of Physics, Yale University, New Haven, CT USA; 232 Yerevan Physics Institute, Yerevan, Armenia; 233 Centre de Calcul de l’Institut National de Physique Nucléaire et de Physique des Particules (IN2P3), Villeurbanne, France; 234 CERN, 1211 Geneva 23, Switzerland

## Abstract

Measurements of the production cross section of a $Z$ boson in association with jets in proton–proton collisions at $\sqrt{s} = 13$ TeV are presented, using data corresponding to an integrated luminosity of 3.16 fb$^{-1}$ collected by the ATLAS experiment at the CERN Large Hadron Collider in 2015. Inclusive and differential cross sections are measured for events containing a $Z$ boson decaying to electrons or muons and produced in association with up to seven jets with $p_{\text {T}} > 30$ GeV and $|y| <2.5$. Predictions from different Monte Carlo generators based on leading-order and next-to-leading-order matrix elements for up to two additional partons interfaced with parton shower and fixed-order predictions at next-to-leading order and next-to-next-to-leading order are compared with the measured cross sections. Good agreement within the uncertainties is observed for most of the modelled quantities, in particular with the generators which use next-to-leading-order matrix elements and the more recent next-to-next-to-leading-order fixed-order predictions.

## Introduction

The measurement of the production of a $Z$ boson^1^ in association with jets, $Z+\text {jets}$, constitutes a powerful test of perturbative quantum chromodynamics (QCD) [1, 2]. The large production cross section and easily identifiable decays of the $Z$ boson to charged leptonic final states offer clean experimental signatures which can be precisely measured. Such processes also constitute a non-negligible background for studies of the Higgs boson and in searches for new phenomena; typically in these studies, the multiplicity and kinematics of the jets are exploited to achieve a separation of the signal of interest from the Standard Model (SM) $Z+\text {jets}$ process. These quantities are often measured in control regions and subsequently extrapolated to the signal region with the use of Monte Carlo (MC) generators, which are themselves subject to systematic uncertainty and must be tuned and validated using data. Predictions from the most recent generators combine next-to-leading-order (NLO) multi-leg matrix elements with a parton shower (PS) and a hadronisation model. Fixed-order parton-level predictions for $Z+\text {jets}$ production at next-to-next-to-leading order (NNLO) are also available [3–6].

The $Z+\text {jets}$ production differential cross section was previously measured by the ATLAS [7], CMS [8], and LHCb [9] collaborations at the CERN Large Hadron Collider (LHC) [10] at centre-of-mass energies of $\sqrt{s}=7$ TeV [11–15] and 8 TeV [16–18], and by the CDF and D0 collaborations at the Tevatron collider at $\sqrt{s} = 1.96$ TeV [19, 20]. In this paper, proton–proton (*pp*) collision data corresponding to an integrated luminosity of 3.16 fb$^{-1}$, collected at $\sqrt{s} = 13$ TeV with the ATLAS detector during 2015, are used for measurements of the $Z$-boson production cross section in association with up to seven jets within a fiducial region defined by the detector acceptance. The $Z$ boson is identified using its decays to electron or muon pairs ($Z \rightarrow e^{+}e^{-}$, $Z \rightarrow \mu ^{+}\mu ^{-}$). Cross sections are measured separately for these two channels, and for their combination, as a function of the inclusive and exclusive jet multiplicity $N_\mathrm {jets}$ and the ratio of successive inclusive jet multiplicities $(N_\mathrm {jets}+1)/N_\mathrm {jets}$, the transverse momentum of the leading jet $p_{\text {T}}^{\text {jet}}$ for several jet multiplicities, the jet rapidity $y^{\text {jet}}$, the azimuthal separation between the two leading jets $\Delta \phi _{\text {jj}}$, the two leading jet invariant mass $m_{\text {jj}}$, and the scalar sum $H_{\text {T}}$ of the transverse momenta of all selected leptons and jets.

The paper is organised as follows. Section 2 contains a brief description of the ATLAS detector. The data and simulated samples as well as the $Z+\text {jets}$ predictions used in the analysis are described in Sect. 3. The event selection and its associated uncertainties are presented in Sect. 4, while the methods employed to estimate the backgrounds are shown in Sect. 5. Comparisons between data and Monte Carlo predictions for reconstructed distributions are found in Sect. 6, while the unfolding procedure is described in Sect. 7. Section 8 presents the analysis results, the comparisons to predictions, and a discussion of their interpretation. Conclusions are provided in Sect. 9.

## The ATLAS detector

The ATLAS experiment at the LHC is a multi-purpose particle detector with a forward-backward symmetric cylindrical geometry and nearly $4\pi$ coverage in solid angle.^2^ It consists of an inner tracking detector, electromagnetic and hadronic calorimeters, and a muon spectrometer. The inner tracker is surrounded by a thin superconducting solenoid magnet and provides precision tracking of charged particles and momentum measurements in the pseudorapidity range $|\eta | < 2.5$. This region is matched to a high-granularity electromagnetic (EM) sampling calorimeter covering the pseudorapidity range $|\eta | < 3.2$, and a coarser granularity calorimeter up to $|\eta | = 4.9$. The hadronic calorimeter system covers the entire pseudorapidity range up to $|\eta | = 4.9$. The muon spectrometer consists of three large superconducting toroids each containing eight coils, a system of trigger chambers, and precision tracking chambers, which provide trigger and tracking capabilities in the range $|\eta | < 2.4$ and $|\eta | < 2.7$, respectively. A two-level trigger system [21] is used to select events. The first-level trigger is implemented in hardware and uses a subset of the detector information. This is followed by the software-based high-level trigger system, which runs offline reconstruction, reducing the event rate to approximately 1 kHz.

## Data set, simulated event samples, and predictions

### Data set

The data used in this analysis were collected by the ATLAS detector during August to November 2015. During this period, the LHC circulated 6.5 $\text {TeV}$proton beams with a 25 ns bunch spacing. The peak delivered instantaneous luminosity was $L={5 \times 10^{33}}$ cm$^{-2}$ s$^{1}$ and the mean number of *pp* interactions per bunch crossing (hard scattering and pile-up events) was $\langle \mu \rangle = 13$. The data set used in this measurement corresponds to a total integrated luminosity of 3.16 fb$^{-1}$.

### Simulated event samples

Monte Carlo simulations, normalised to higher-order calculations, are used to estimate most of the contributions from background events, to unfold the data to the particle level, and to compare with the unfolded data distributions. All samples are processed with a Geant4-based simulation [22] of the ATLAS detector [23]. An overview of all signal and background processes considered and of the generators used for the simulation is given in Table 1. Total production cross sections for the samples, their respective uncertainties (mainly coming from parton distribution function (PDF) and factorisation and renormalisation scale variations), and references to higher-order QCD corrections, where available, are also listed in Table 1.

**Table 1:** Signal and background Monte Carlo samples and the generators used in the simulation. Each sample is normalised to the appropriate production cross section $\sigma$ and multiplied by the relevant branching ratios (${\mathrm {BR}}$) per lepton flavour for this sample, as shown in the third column. For $W$-boson and top-quark production, contributions from higher-order QCD corrections were calculated following the references given in the fifth column for the stated order. Similarly, for $Z$-boson production, higher-order QCD corrections were evaluated in the dilepton invariant mass range $66<m_{\ell \ell }<116$ GeV following the references given in the fifth column for the stated order, and extrapolation scaling factors were applied to match mass ranges used by each simulation as given in the first column. The theory uncertainties as given in the final column correspond to PDF and scale variations. The diboson samples include on-shell and off-shell *WW*, *WZ* and *ZZ* production. Recently, NNLO QCD predictions have been made available for the diboson processes [32, 33]. However, these higher-order corrections have a negligible impact on this analysis

Process	Generator	$(\sigma \cdot \mathrm {BR})$ [pb]	Normalisation order	References	Theory uncert. (%)
$Z (\rightarrow \ell ^{+}\ell ^{-})+\text {~jets}$ ($\ell = e,\mu ; m_{\ell \ell }>40$ GeV)	Sherpa 2.2	2106	NNLO	[24–27]	5
$Z (\rightarrow \ell ^{+}\ell ^{-})+\text {~jets}$ ($\ell = e,\mu ,\tau ; m_{\ell \ell }>40$ GeV)	MG5_aMC@NLO+Py8	2103	NNLO	[24–27]	5
$W \rightarrow \ell \nu$ ($\ell = e,\mu$)	MG5_aMC@NLO+Py8	20,080	NNLO	[24–27]	5
$t\overline{t}$ ($m_{t}=172.5$ GeV)					
Perugia2012(radHi/radLo)	Powheg+Py6	831	NNLO+NNLL	[28]	6
UE-EE-5	MG5_aMC@NLO+Herwig++	831	NNLO+NNLL	[28]	6
Single top quark (*Wt*)	Powheg+Py6	72	NLO+NNLL	[29]	6
Single top quark (*t*-channel)	Powheg+Py6	136	NLO+NNLL	[30]	6
Single top anti-quark(*t*-channel)	Powheg+Py6	81	NLO+NNLL	[30]	6
Dibosons	Sherpa 2.1	97	NLO	[31]	6

Signal events (i.e. containing a $Z$ boson with associated jets) are simulated using the Sherpa  v2.2.1 [31] generator, denoted by Sherpa 2.2. Matrix elements (ME) are calculated for up to two additional partons at NLO and up to four partons at leading order (LO) using the Comix [34] and OpenLoops [35] matrix element generators. They are merged with the Sherpa parton shower [36] (with a matching scale of 20 GeV) using the ME+PS@NLO prescription [37]. A five-flavour scheme is used for these predictions. The NNPDF30NLO PDF set [38] is used in conjunction with a dedicated set of parton-shower-generator parameters (tune) developed by the Sherpa authors. This sample is used for the nominal unfolding of the data distributions, to compare to the cross-section measurements, and to estimate the systematic uncertainties.

A simulated sample of $Z+\text {jets}$ production is also produced with the MADGRAPH_aMC@NLO (denoted by MG5_aMC@NLO) v2.2.2 generator [39], using matrix elements including up to four partons at leading order and employing the NNPDF30NLO PDF set, interfaced to Pythia  v8.186 [40] to model the parton shower, using the CKKWL merging scheme [41] (with a matching scale of 30 GeV). A five-flavour scheme is used. The A14 [42] parton-shower tune is used together with the NNPDF23LO PDF set [43]. The sample is denoted by MG5_aMC+Py8 CKKWL and is used to provide cross-checks of the systematic uncertainty in the unfolding and to model the small $Z \rightarrow \tau \tau$ background. In addition, a MG5_aMC@NLO sample with matrix elements for up to two jets and with parton showers beyond this, employing the NNPDF30NLO PDF set and interfaced to Pythia  v8.186 to model the parton shower, is generated using the FxFx merging scheme [44] (with a matching scale of 25 GeV [45]) and is denoted by MG5_aMC+Py8 FxFx. This sample also uses a five-flavour scheme and the A14 parton-shower tune with the NNPDF23LO PDF set. Both MG5_aMC@NLO samples are used for comparison with the unfolded cross-section measurements.

The measured cross sections are also compared to predictions from the leading-order matrix element generator Alpgen v2.14 [46] interfaced to Pythia  v6.426 [47] to model the parton shower, denoted by Alpgen+Py6, using the Perugia2011C [48] parton-shower tune and the CTEQ6L1 PDF set [49]. A four-flavour scheme is used. Up to five additional partons are modelled by the matrix elements merged with the MLM prescription [46] (with a matching scale of 20 GeV). The matrix elements for the production of $Z+b\bar{b}$ and $Z+c\bar{c}$ events are explicitly included and a heavy-flavour overlap procedure is used to remove the double counting of heavy quarks from gluon splitting in the parton shower.

The $Z$-boson samples are normalised to the NNLO prediction calculated with the Fewz 3.1 program [24–27] with CT10nnlo PDFs [50].

Contributions from the top-quark, single-boson, and diboson components of the background (described in Sect. 5) are estimated from the following Monte Carlo samples. Samples of top-quark pair and single top-quark production are generated at NLO with the Powheg-Box generator [51–53] [versions v2 (r3026) for top-quark pairs and v1 for single top quarks (r2556 and r2819 for *t*-and *Wt*-channels, respectively)] and Pythia  v6.428 (Perugia2012 tune [48]). Samples with enhanced or suppressed additional radiation are generated with the Perugia2012radHi/Lo tunes [48]. An alternative top-quark pair sample is produced using the MG5_aMC@NLO generator interfaced with Herwig++  v2.7.1 [39, 54], using the UE-EE-5 tune [55]. The samples are normalised to the cross section calculated at NNLO+NNLL (next-to-next-to-leading log) with the Top++2.0 program [28].

The $W$-boson backgrounds are modelled using the MG5_aMC+Py8 CKKWL  v2.2.2 generator, interfaced to Pythia  v8.186 and are normalised to the NNLO values given in Table 1. Diboson processes with fully leptonic and semileptonic decays are simulated [56] using the Sherpa  v2.1.1 generator with the CT10nlo PDF set. The matrix elements contain the doubly resonant *WW*, *WZ* and *ZZ* processes, and all other diagrams with four electroweak vertices. They are calculated for one or zero additional partons at NLO and up to three additional partons at LO and merged with the Sherpa parton shower using the ME+PS@NLO prescription.

Events involving semileptonic decays of heavy quarks, hadrons misidentified as leptons, and, in the case of the electron channel, electrons from photon conversions are referred to collectively as “multijet events”. The multijet background was estimated using data-driven techniques, as described in Sect. 5.

Multiple overlaid *pp* collisions are simulated with the soft QCD processes of Pythia  v.8.186 using the A2 tune [57] and the MSTW2008LO PDF set [58]. All Monte Carlo samples are reweighted so that the pile-up distribution matches that observed in the data.

### Fixed-order predictions

In addition to these Monte Carlo samples, parton-level fixed-order predictions at NLO are calculated by the BlackHat+Sherpa collaboration for the production of $Z$ bosons with up to four partons [59, 60]. The BlackHat+Sherpa predictions use the CT14 PDF set [61] including variations of its eigenvectors at the 68% confidence level, rescaled from 90% confidence level using a factor of 1 / 1.645. The nominal predictions use a factorisation and renormalisation scale of $H_{\text {T}}/2$ with uncertainties derived from the envelope of a common variation of the scales by factors of $0.5, 1/\sqrt{2}, \sqrt{2},$ and 2. The effects of PDF and scale uncertainties range from 1 to 4% and from 0.1 to 10%, respectively, for the cross sections of $Z$-boson production in association with at least one to four partons, and are included in the predictions which are provided by the BlackHat+Sherpa authors for the fiducial phase space of this analysis. Since these predictions are defined before the decay leptons emit photons via final-state radiation (Born-level FSR), corrections to the dressed level (where all photons found within a cone of size $\Delta R = 0.1$ of the lepton from the decay of the $Z$ boson are included) are derived from MG5_aMC+Py8 CKKWL, separately for each kinematic observable used to measure cross sections, with associated systematic uncertainties obtained by comparing to the Alpgen+Py6 generator. This correction is needed in order to match the prediction to the lepton definition used in the measurements. The average size of these corrections is approximately $-2$%. To bring the prediction from parton to particle level, corrections for the non-perturbative effects of hadronisation and the underlying event are also calculated separately for each observable using the Sherpa  v2.2 generator by turning on and off in the simulation both the fragmentation and the interactions between the proton remnants. The net size of the corrections is up to approximately 10% at small values of $p_{\text {T}}^{\text {jet}}$ and vanishes for large values of $p_{\text {T}}^{\text {jet}}$. An uncertainty of approximately 2% for this correction is included in the total systematic uncertainty of the prediction.

Calculations of cross sections at NNLO QCD have recently become available [3–6]. In this paper, the results are compared to the calculation, denoted by $Z+ \ge 1\text {~jet~N}_{\text {jetti}}$ NNLO [3, 4], which uses a new subtraction technique based on *N*-*jettiness* [62] and relies on the theoretical formalism provided in soft-collinear effective theory. The predictions, which are provided by the authors of this calculation for the fiducial phase space of this analysis, use a factorisation and renormalisation scale of $\sqrt{m_{\ell \ell }^2 + \sum \left( p_{\text {T}}^{\text {jet}}\right) ^2}$ (where $m_{\ell \ell }$ is the invariant mass of the dilepton system) and the CT14 PDF set. The QCD renormalisation and factorisation scales were jointly varied by a common factor of two, and are included in the uncertainties. Non-perturbative and FSR corrections and their associated uncertainties as discussed above are also included in the predictions.

## Event selection

Electron- and muon-candidate events are selected using triggers which require at least one electron or muon with transverse momentum thresholds of $p_{\text {T}} =24\,\text {GeV}$ or 20 $\text {GeV}$, respectively, with some isolation requirements for the muon trigger. To recover possible efficiency losses at high momenta, additional electron and muon triggers which do not make any isolation requirements are included with thresholds of $p_{\text {T}} \ge 60\,\text {GeV}$ and $p_{\text {T}} = 50\,\text {GeV}$, respectively. Candidate events are required to have a primary vertex, defined as the vertex with the highest sum of track $p_{\text {T}} ^2$, with at least two associated tracks with $p_{\text {T}} > 400\,\text {MeV}$.

Electron candidates are required to have $p_{\text {T}} > 25\,\text {GeV}$ and to pass “medium” likelihood-based identification requirements [63, 64] optimised for the 2015 operating conditions, within the fiducial region of $|\eta |<2.47$, excluding candidates in the transition region between the barrel and endcap electromagnetic calorimeters, $1.37<|\eta |<1.52$. Muons are reconstructed for $|\eta |<2.4$ with $p_{\text {T}} > 25\,\text {GeV}$ and must pass “medium” identification requirements [65] also optimised for the 2015 operating conditions. At least one of the lepton candidates is required to match the lepton that triggered the event. The electrons and muons must also satisfy $p_{\text {T}}$-dependent cone-based isolation requirements, using both tracking detector and calorimeter information (described in Refs. [66, 67], respectively). The isolation requirements are tuned so that the lepton isolation efficiency is at least 90% for $p_{\text {T}} >25\,\text {GeV}$, increasing to 99% at 60 GeV. Both the electron and muon tracks are required to be associated with the primary vertex, using constraints on the transverse impact parameter significance $|d_0|/\Delta d_0$, where $d_0$ is the transverse impact parameter and $\Delta d_0$ is its uncertainty, and on the longitudinal impact parameter $z_0$ corrected for the reconstructed position of the primary vertex. The transverse impact parameter significance is required to be less than five for electrons and three for muons, while the absolute value of the corrected $z_0$ multiplied by the sine of the track polar angle is required to be less than 0.5 mm.

Jets of hadrons are reconstructed with the anti-$k_t$ algorithm [68] with radius parameter $R=0.4$ using topological clusters of energy deposited in the calorimeters [69]. Jets are calibrated using a simulation-based calibration scheme, followed by in situ corrections to account for differences between simulation and data [70]. In order to reduce the effects of pile-up contributions, jets with pseudorapidity $|\eta |<2.4$ and $p_{\text {T}} <{60} {\text {GeV}}$ are required to have a significant fraction of their tracks with an origin compatible with the primary vertex, as defined by the jet vertex tagger algorithm [71]. In addition, the expected average energy contribution from pile-up clusters is subtracted according to the $\eta$–$\phi$ catchment area of the jet [72]. Jets used in the analysis are required to have $p_{\text {T}}$ greater than 30 GeV and rapidity $|y|<2.5$.

The overlap between leptons and jets is removed in a two-step process. The first step removes jets closer than $\Delta R = 0.2$ to a selected electron, and jets closer than $\Delta R = 0.2$ to a selected muon, if they are likely to be reconstructed from photons radiated by the muon. In a second step, electrons and muons are discarded if they are located closer than $\Delta R = 0.4$ to a remaining selected jet. This requirement effectively removes events with leptons and jets which are not reliably simulated in the Monte Carlo simulation.

Events containing a $Z$-boson candidate are selected by requiring exactly two leptons of the same flavour but of opposite charge with dilepton invariant mass in the range $71< m_{\ell \ell } < 111$ GeV. The expected and observed numbers of $Z$-boson candidates selected for each inclusive jet multiplicity, for $N_{\mathrm {jets}} \ge 0-7$, are summarised in Table 2, separately for the $Z \rightarrow e^{+}e^{-}$ and the $Z \rightarrow \mu ^{+}\mu ^{-}$ channels. The background evaluation that appears in this table is discussed in Sect. 5. After all requirements, 248,816 and 311,183 $Z+ \ge 1\text {~jet}$ events are selected in the electron and muon channels, respectively.

**Table 2:** Fraction of signal and background processes in % in the final selection and expected and observed numbers of events for the various inclusive jet multiplicities considered in the electron (top) and muon (bottom) channels

	$\texttt {+}\ge \mathrm {0~jets}$	$\texttt {+}\ge \mathrm {1~jet}$	$\texttt {+}\ge \mathrm {2~jets}$	$\texttt {+}\ge \mathrm {3~jets}$	$\texttt {+}\ge \mathrm {4~jets}$	$\texttt {+}\ge \mathrm {5~jets}$	$\texttt {+}\ge \mathrm {6~jets}$	$\texttt {+}\ge \mathrm {7~jets}$
Electron channel								
$Z \rightarrow e^{+}e^{-}$ (%)	99.3	97.6	93.9	90.3	87.3	85.2	83.3	81.2
Top quark (%)	0.2	1.2	3.8	6.5	8.6	9.7	10.5	11.6
Diboson (%)	0.2	0.8	1.6	2.4	3.4	4.4	5.5	6.6
$Z\rightarrow \tau ^{+}\tau ^{-}$ (%)	${<}$0.1	${<}$0.1	${<}$0.1	${<}$0.1	${<}$0.1	${<}$0.1	${<}$0.1	${<}$0.1
$W \rightarrow e\nu$ (%)	${<}$0.1	${<}$0.1	${<}$0.1	${<}$0.1	${<}$0.1	${<}$0.1	${<}$0.1	${<}$0.1
Multijet (%)	0.2	0.4	0.6	0.7	0.7	0.7	0.7	0.7
Expected	1,327,900	239,500	57,310	14,080	3637	978	252	63
Observed	1,347,900	248,816	59,998	14,377	3587	898	217	48
Muon channel								
$Z \rightarrow \mu ^{+}\mu ^{-}$ (%)	99.3	97.5	94.0	90.7	88.3	86.7	84.8	84.6
Top quark (%)	0.2	1.1	3.6	6.0	7.7	8.1	8.7	7.7
Diboson (%)	0.2	0.7	1.6	2.4	3.4	4.5	5.9	7.0
$Z\rightarrow \tau ^{+}\tau ^{-}$ (%)	${<}$0.1	${<}$0.1	${<}$0.1	${<}$0.1	${<}$0.1	${<}$0.1	${<}$0.1	${<}$0.1
$W \rightarrow \mu \nu$ (%)	${<}$0.1	${<}$0.1	${<}$0.1	${<}$0.1	${<}$0.1	${<}$0.1	${<}$0.1	${<}$0.1
Multijet (%)	0.3	0.6	0.9	0.9	0.7	0.7	0.7	0.7
Expected	1,693,000	300,600	71,230	17,740	4523	1187	307	76
Observed	1,708,602	311,183	74,510	17,865	4387	1081	240	57

### Correction factors and related systematic uncertainties

Some of the object and event selection efficiencies as well as the energy and momentum calibrations modelled by the simulation must be corrected with simulation-to-data correction factors to better match those observed in the data. These corrections and their corresponding uncertainties fall into the following two categories: dependent and not dependent on lepton flavour.

The corrections and uncertainties specific to each leptonic final state ($Z \rightarrow e^{+}e^{-}$ and $Z \rightarrow \mu ^{+}\mu ^{-}$) are as follows:

- **Trigger:** The lepton trigger efficiency is estimated in simulation, with a separate data-driven analysis performed to obtain the simulation-to-data trigger correction factors and their corresponding uncertainties [21].
- **Lepton reconstruction, identification, and isolation:** The lepton selection efficiencies as determined from simulation are also corrected with simulation-to-data correction factors, with corresponding uncertainties [64, 65].
- **Energy, momentum scale/resolution:** Uncertainties in the lepton calibrations are estimated [65] because they can cause a change of acceptance because of migration of events across the $p_{\text {T}}$ threshold and $m_{\ell \ell }$ boundaries.

The corrections and uncertainties common to the electron and muon final states are as follows:

- **Jet energy scale and resolution**: Uncertainties in the jet energy-scale calibration and resolution have a significant impact on the measurements, especially for the higher jet multiplicities. The jet energy-scale calibration is based on 13 TeV simulation and on in situ corrections obtained from data [70]. The uncertainties are estimated using a decorrelation scheme, resulting in a set of 19 independent parameters which cover all of the relevant calibration uncertainties. The jet energy scale is the dominant systematic uncertainty for all bins with at least one jet. The jet energy-resolution uncertainty is derived by over-smearing the jet energy in the simulation and using the symmetrised variations as the uncertainty.
- **Jet vertex tagger**: The modelling of the output variable from the jet vertex tagger is validated using data events where the $Z$ boson recoils against a jet. A percent-level correction is derived and its statistical and systematic uncertainties are used as additional uncertainties in the efficiency to select jets from the primary vertex [71].
- **Pile-up**: The imperfect modelling of the effects of pile-up leads to acceptance changes at the percent level for different jet multiplicities. To assess this uncertainty, the average number of interactions per bunch crossing $\langle \mu \rangle$ is varied in simulation so that the behaviour of variables sensitive to pile-up matches that observed in data.
- **Luminosity**: The cross sections have a 2.1% uncertainty from the measurement of the integrated luminosity, which is derived, following a methodology similar to that detailed in Refs. [73, 74], from a calibration of the luminosity using *x*–*y* beam-separation scans performed in August 2015.

## Background estimation

Contributions from the electroweak (single boson and diboson) and top-quark (single top-quark and top-quark pair) components of the background are estimated using the Monte Carlo samples described in Sect. 3 with corresponding uncertainties as listed in Table 1. Contributions from multijet events are evaluated with data-driven techniques as described below. A summary of the composition and relative importance of the backgrounds in the candidate $Z+\text {jets}$ events is given in Table 2. The overall purity of the $Z+\text {jets}$ selections (fraction of signal events in the final selection) ranges from 99% in the inclusive sample to 80–85% in the $\ge \mathrm {7~jets}$ bin.

### Top-quark and electroweak backgrounds

The dominant contribution to the background at high jet multiplicities comes from $t\overline{t}$ production, with the subsequent leptonic decays of the $W$ bosons originating from the top quarks and is evaluated from simulation. An overall uncertainty of 6%, corresponding to the PDF and scale variations on the theoretical predictions of the inclusive cross sections, is assigned (see Table 1). The $t\overline{t}$ background estimate is validated through a cross-section measurement of $t\overline{t}$ production in the dilepton channel at $\sqrt{s} = 13$ TeV [75] as a function of the jet multiplicity, and the modelling of the additional parton radiation in $t\overline{t}$ events by Powheg+Py6 was found to be in good agreement with this measurement. In addition, a systematic uncertainty in the modelling of the shape of the distributions is derived by modifying the parton-shower intensity in the nominal simulation sample and by comparing to the predictions from the alternative generator MG5_aMC@NLO+Herwig++ (both listed in Table 1). The small contribution from single-top-quark events is also estimated using Powheg+Py6 samples and assigned a 6% uncertainty.

Diboson production in leptonic and semileptonic final states with at least two leptons of the same flavour constitutes a co-dominant background for high jet multiplicities (see Table 2). The production of *WZ* bosons in association with jets at $\sqrt{s}=13$ TeV was found to be well modelled by the Sherpa 2.1 generator [76]. A 6% uncertainty, again corresponding to PDF and scale variations on the predictions, is assessed. Since in Ref. [76] the measurement is limited by the statistical precision for dibosons $+\ge 4$ jets (resulting in $\ge 6$ hadronic jets for semileptonic diboson decays), an additional systematic uncertainty of 50% in the normalisation of the diboson background is added for $Z+ \ge 6\text {~jets}$.

Minor background contributions also arise from single-$W$-boson production decaying to leptonic final states and from single-$Z$-boson production in the $Z \rightarrow \tau ^{+}\tau ^{-}$ process, both estimated with simulation and assigned a 5% uncertainty (as given in Table 1).

### Multijet background

Background-enriched data control regions are used to estimate the multijet contribution in both the electron and muon channels. They are constructed by loosening the lepton identification and isolation requirements. Templates are built from the dilepton invariant mass distribution, a variable that shows discrimination between multijet background and other processes in regions of its kinematic range, but is largely uncorrelated with the variables used to build the multijet control regions. The templates are subsequently normalised to events passing the $Z$-boson signal selection.

In the electron channel, the multijet templates are built for each jet multiplicity from events with two same-charge leptons with no isolation requirement, whose identification criteria are looser than those of the signal selection, which the leptons must not satisfy. In the muon channel, the control region is similarly built from events with two leptons which are selected with looser identification requirements than the signal selection and also fail the nominal isolation requirement. In both cases, dedicated triggers better suited to this purpose are used to populate the templates. The small electroweak and top-quark contamination is subtracted using simulated events.

The normalisation of the multijet template is estimated with a log-likelihood fit to the measured dilepton invariant mass distribution for the inclusive $Z$ selection, using templates for $Z \rightarrow \ell ^{+}\ell ^{-}$ and for the electroweak and top-quark background derived from simulation. The fit is performed in the invariant mass windows of $52< m_{ee} < 148$ GeV and $40< m_{\mu \mu } < 80$ GeV for the electron and muon channels, respectively, in order to benefit from the larger multijet contribution in the mass sidebands. The normalisation of the multijet template is allowed to float freely while the remaining non-multijet templates are constrained to be within 6% of the predicted cross sections for these processes as given in Table 1. The multijet fractions are evaluated separately for each jet multiplicity, except for very high jet multiplicities where the templates are statistically limited, and so these fractions are taken from the estimates of the $\ge \mathrm {5~jets}$ and $\ge \mathrm {4~jets}$ bins in the electron and muon channels, respectively.

The systematic uncertainties on the multijet background are derived by varying the mass range and bin width of the nominal fit, using the lepton transverse impact parameter $d_{0}$ as the fitting variable instead of the invariant mass, using alternative simulation samples for the templates, allowing the normalisations of the non-multijet components to vary independently or within a wider range, and varying the lepton resolution and energy/momentum scales. In addition, given the multiple sources of multijet background in the electron channel, an alternative template is constructed by requiring that the electrons fail to meet an isolation criterion instead of failing to meet the nominal signal selection electron identification criterion.

The resulting estimated multijet fractions in each jet multiplicity bin are given in Table 2. Their corresponding total uncertainties are dominated by their systematic components. These systematic components are approximately 70% of the multijet fraction as estimated in the electron and muon channels.

## Kinematic distributions

**Fig. 1:**
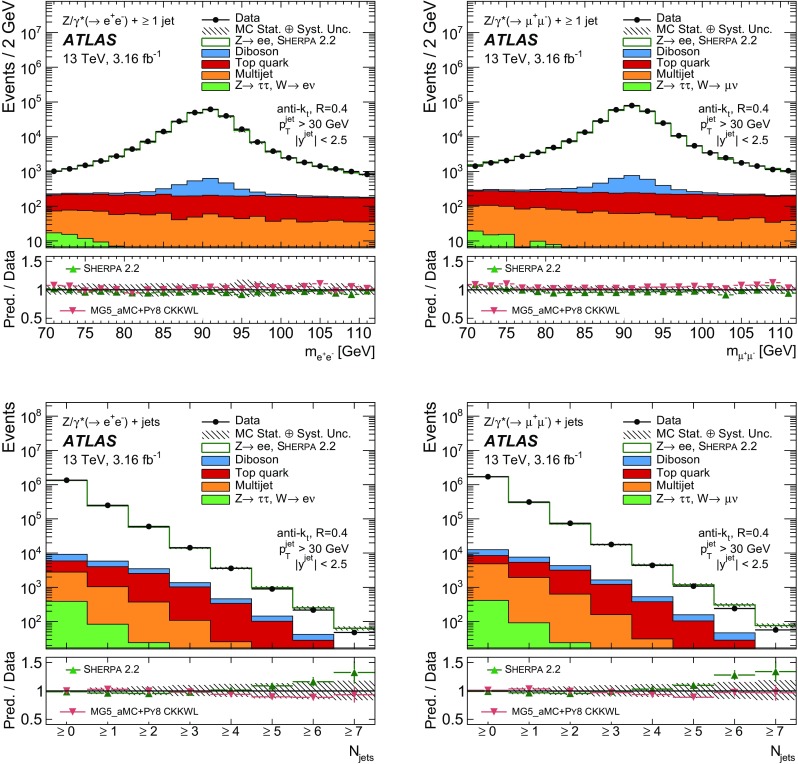
Dilepton invariant mass for $Z+ \ge 1\text {~jet}$ (*top*) and inclusive jet multiplicity (*bottom*) in the $Z (\rightarrow e^{+}e^{-})+\text {~jets}$ (*left*) and the $Z (\rightarrow \mu ^{+}\mu ^{-})+\text {~jets}$ (*right*) channels. All backgrounds and the signal samples are stacked to produce the figures. Systematic uncertainties for the signal and background distributions are combined in the *hatched band*, and the statistical uncertainty is shown on the *data points*. The uncertainty in the luminosity and the theory uncertainty in the signal prediction are not included in the *uncertainty band*

The level of agreement between data and predictions is evaluated from the comparison of kinematic distributions. Figure 1, which presents the dilepton mass for the $Z+ \ge 1\text {~jet}$ topology and the inclusive jet multiplicity, shows how well the Sherpa 2.2 and MG5_aMC+Py8 CKKWL predictions agree with data. The uncertainty bands shown in these distributions include the statistical uncertainties due to the simulation sample sizes, the event-selection uncertainties described in Sect. 4.1 (omitting the common 2.1% luminosity uncertainty), and the background normalisation uncertainties described in Sect. 5.

## Unfolding of detector effects

The cross-section measurements presented in this paper are performed within the fiducial acceptance region defined by the following requirements:

- $$p_{\text {T}} ^{\ell }>25$$ GeV, $$|\eta ^{\ell }|<2.5$$
- $$p_{\text {T}}^{\text {jet}} > 30$$ GeV, $$|y^{\text {jet}} |<2.5$$
- $$\Delta R(\ell ,\text {jet}) > 0.4$$
- $$71<m_{\ell \ell }<111$$ GeV.

The cross sections are defined at particle (“truth”) level, corresponding to dressed electrons and muons from the $Z$ bosons. The particle level also includes jets clustered using the anti-$k_t$ algorithm [68] with radius parameter $R=0.4$ for final-state particles with decay length $c\tau >10$ mm, excluding the dressed $Z$-boson decay products.

The fiducial cross sections are estimated from the reconstructed kinematic observables: jet multiplicity, $p_{\text {T}}^{\text {jet}}$ for different jet multiplicities, $y^{\text {jet}}$, $\Delta \phi _{\text {jj}}$, $m_{\text {jj}}$, and $H_{\text {T}}$, for events that pass the selection described in Sect. 4. The expected background components as described in Sect. 5 are subtracted from the distributions in data. A variable-width binning of these observables is used, such that the purity is at least 50% in each bin and the size of the statistical uncertainty in most of the bins remains below 10%.

**Fig. 2:**
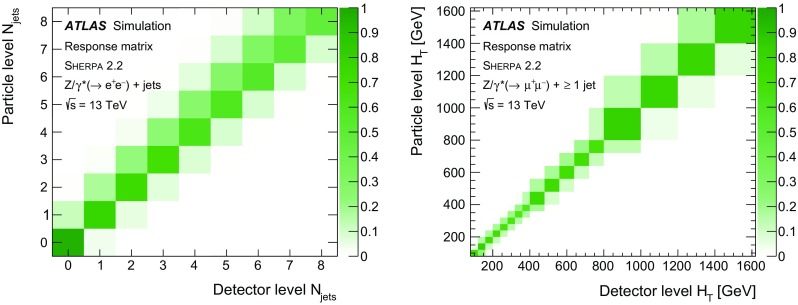
Response matrices corresponding to the exclusive jet multiplicity for $Z+\text {jets}$ events in the electron channel (*left*) and to the $H_{\text {T}}$ for $Z+ \ge 1\text {~jet}$ events in the muon channel (*right*). The sum of the entries in each row is normalised to unity. Both matrices are obtained from Sherpa 2.2

An iterative Bayesian unfolding technique [77], as implemented in the RooUnfold package [78], is used to unfold the measurements to the particle level, thereby accounting for detector effects related to inefficiencies, resolution, and systematic biases in the central values of the kinematic variables describing both the leptons and the jets. The iterative unfolding technique updates the initial estimators for the generated (“truth”) distribution in consecutive steps, using Bayes’ theorem in each iteration to derive an unfolding matrix from the initial response matrix (which relates truth and reconstructed distributions of given observables) and the current truth estimator.

The response matrices are constructed using the Sherpa 2.2 $Z (\rightarrow \ell ^{+}\ell ^{-})+\text {~jets}$ samples. Sherpa 2.2 is also used to derive the initial truth estimator. In order to enter the response matrix, events must pass the $Z$-boson selection at generator level and at detector level and contain the number of jets required by the preselection for a given observable at both generator and detector level. Reconstructed jets are required to match the corresponding generator-level jets within a cone of size $\Delta R=0.4$ for all distributions except global quantities such as the jet multiplicity and $H_{\text {T}}$. A given bin (*i*, *j*) in the response matrix therefore corresponds to the probability that a true jet object in bin *j* is reconstructed in bin *i* of the distribution. Figure 2 illustrates two examples of response matrices. The resulting ratios of detector-level to truth-level event yields are typically 0.65 and 0.8 for the electron and muon channels, respectively.

The background-subtracted data are corrected for the expected fraction of events with reconstructed objects unmatched to any generator object before entering the iterative unfolding. The number of iterations used for the iterative unfolding of each distribution (two) is chosen by unfolding the Sherpa 2.2 samples reweighted to data and comparing to the generated reweighted distribution. The unfolded event yields are divided by the integrated luminosity of the data sample and the bin width of the distribution in question to provide the final fiducial cross sections. The final result is given by

1 $$\begin{aligned} \sigma _i = \frac{1}{\epsilon _i L} \sum _j U_{ij} N_j^{\text {data}} \left( 1-f_j^{\text {unmatched}}\right) , \end{aligned}$$

where *L* is the integrated luminosity, $\epsilon _i$ is the reconstruction efficiency for truth bin *i*, $N_j^{\text {data}}$ corresponds to the number of events observed in data in reconstructed bin *j* and $f_j^{\text {unmatched}}$ is its fraction of unmatched events calculated from simulation, and $U_{ij}$ is the unfolding matrix calculated after two iterations, using the updated prior from the first iteration and the response matrix.

### Systematic uncertainties associated with the unfolding procedure

The limited size of a simulation sample can create biases in the distributions. Systematic uncertainties account for possible residual biases in the unfolding procedure due to, e.g. modelling of the hadronisation in the simulation, migrations into other kinematic distributions not explicitly part of the unfolding, or the finite bin width used in each distribution. The following uncertainties arise from the unfolding procedure.

- The statistical uncertainties of the response matrices derived from Sherpa 2.2 are propagated to the unfolded cross sections with a toy simulation method. A total of 5000 ensembles (pseudo-experiments) of unfolded samples are generated. For each sample, the number of reconstructed events in each bin is generated randomly according to a Gaussian distribution, where the mean is the nominal number of events before unfolding and the width is its corresponding statistical uncertainty. Unfolding is performed for each ensemble. The widths of resulting distributions are taken as a systematic uncertainty of the unfolding.
- The Sherpa 2.2 samples are reweighted at generator level, such that the distribution of the leading jet $p_{\text {T}}$ at detector level matches that observed in the data. The modified Sherpa 2.2 samples are then used to unfold the data again and the variations in the resulting cross sections are used to derive a systematic uncertainty.
- An additional check is performed by unfolding reconstructed MG5_aMC+Py8 CKKWL events using Sherpa 2.2 response matrices. The residual non-closure is accounted for by an additional flat uncertainty of 3% for all distributions.

## Results

The measured cross sections, presented in Sect. 8.1, are calculated in the electron and muon channels separately and the compatibility of the results of the two channels is evaluated. In order to improve the precision of the measurement, these results are then combined, taking into account the correlations of the systematic uncertainties. The comparisons of the combined results to the predictions are presented in Sect. 8.2.

### Results in the individual channels and the combination

The fiducial cross-section measurements in the $Z (\rightarrow e^{+}e^{-})+\text {~jets}$ and $Z (\rightarrow \mu ^{+}\mu ^{-})+\text {~jets}$ channels as a function of the inclusive jet multiplicities are presented in Table 3. The data statistical uncertainties are propagated through the unfolding by using pseudo-experiments. As mentioned in Sect. 7, the systematic uncertainties are propagated through the unfolding via the migration matrices and via the variation of the subtracted background. Table 4 shows the resulting total relative statistical and systematic uncertainties as well as the systematic components [lepton trigger, lepton selection, jet energy scale and resolution, jet vertex tagging, pile-up, luminosity (all described in Sect. 4.1)], unfolding (described in Sect. 7), and background (described in Sect. 5) as a function of the inclusive jet multiplicity, presented separately for the electron and muon channels. The jet energy scale is the dominant systematic uncertainty for all bins with at least one jet.

**Table 3:** Measured fiducial cross sections in the electron and muon channels for successive inclusive jet multiplicities. The total statistical and systematic uncertainties are given, along with the uncertainty in the luminosity

Jet multiplicity	Measured cross section ± (stat.) ± (syst.) ± (lumi.) [pb]							
	$Z \rightarrow ee$				$Z \rightarrow \mu \mu$			
${\ge }$0 jets	743${\pm }$	1${\pm }$	24${\pm }$	16	738${\pm }$	1${\pm }$	23${\pm }$	16
${\ge }$1 jets	116.6 ${\pm }$	0.3${\pm }$	9.9${\pm }$	2.5	115.7${\pm }$	0.2${\pm }$	9.7${\pm }$	2.5
${\ge }$2 jets	27.1${\pm }$	0.1${\pm }$	2.9${\pm }$	0.6	27.0${\pm }$	0.1${\pm }$	2.8${\pm }$	0.6
${\ge }$3 jets	6.20${\pm }$	0.06${\pm }$	0.82${\pm }$	0.14	6.22${\pm }$	0.05${\pm }$	0.83${\pm }$	0.14
${\ge }$4 jets	1.49${\pm }$	0.03${\pm }$	0.23${\pm }$	0.04	1.48${\pm }$	0.03${\pm }$	0.23${\pm }$	0.04
${\ge }$5 jets	0.357${\pm }$	0.013${\pm }$	0.069${\pm }$	0.009	0.354 ${\pm }$	0.012 ${\pm }$	0.068${\pm }$	0.009
${\ge }$6 jets	0.082${\pm }$	0.006${\pm }$	0.019${\pm }$	0.002	0.076 ${\pm }$	0.005${\pm }$	0.019${\pm }$	0.002
${\ge }$7 jets	0.0180${\pm }$	0.0029${\pm }$	0.0051${\pm }$	0.0005	0.0166${\pm }$	0.0027${\pm }$	0.0060${\pm }$	0.0004

**Table 4:** Relative statistical and systematic uncertainties (in %) in the measured cross sections of $Z+\text {jets}$ production for successive inclusive jet multiplicities in the electron (top) and muon (bottom) channels

Systematic source	Relative uncertainty in $\sigma (Z (\rightarrow \ell ^{+}\ell ^{-})+\ge {N_{\mathrm jets}})$ (%)							
	+ $\ge$ 0 jet	+ $\ge$ 1 jet	+ $\ge$ 2 jets	+ $\ge$ 3 jets	+ $\ge$ 4 jets	+ $\ge$ 5 jets	+ $\ge$ 6 jets	+ $\ge$ 7 jets
$$Z \rightarrow e^{+}e^{-}$$								
Electron trigger	0.1	0.1	0.1	0.2	0.2	0.2	0.3	0.3
Electron selection	1.2	1.6	1.8	1.9	2.3	2.7	2.9	3.8
Jet energy scale	${<}$0.1	6.6	9.2	11.5	13.8	17.3	20.6	23.7
Jet energy resolution	${<}$0.1	3.7	3.7	4.4	5.3	5.2	6.2	7.3
Jet vertex tagger	${<}$0.1	1.3	2.1	2.8	3.6	4.5	5.5	6.3
Pile-up	0.4	0.2	0.1	0.2	0.2	0.1	0.4	0.8
Luminosity	2.1	2.1	2.2	2.3	2.4	2.5	2.6	2.8
Unfolding	3.0	3.0	3.0	3.0	3.0	3.1	3.1	3.2
Background	0.1	0.3	0.6	1.0	1.6	3.3	6.0	11.6
Total syst. Uncertainty	3.9	8.7	11.0	13.4	15.9	19.5	23.6	28.7
Stat. uncertainty	0.1	0.2	0.5	0.9	1.9	3.7	7.7	15.9
$$Z \rightarrow \mu ^{+}\mu ^{-}$$								
Muon trigger	0.4	0.5	0.4	0.5	0.4	0.5	0.9	0.6
Muon selection	0.8	0.9	1.0	1.0	1.0	1.5	4.2	16.6
Jet energy scale	${<}$0.1	6.8	9.1	11.9	14.0	17.0	20.9	23.7
Jet energy resolution	${<}$0.1	3.6	3.6	4.1	5.0	5.9	6.2	9.3
Jet vertex tagger	${<}$0.1	1.3	2.1	3.1	3.6	4.4	5.6	6.6
Pile-up	0.4	0.1	0.0	0.3	0.5	0.1	0.4	0.9
Luminosity	2.1	2.1	2.2	2.3	2.4	2.5	2.6	2.7
Unfolding	3.0	3.0	3.0	3.0	3.0	3.1	3.1	3.2
Background	0.2	0.4	0.6	0.9	1.7	4.0	7.4	12.9
Total syst. Uncertainty	3.8	8.7	10.8	13.6	16.0	19.4	24.6	36.3
Stat. uncertainty	0.1	0.2	0.4	0.8	1.7	3.4	7.2	16.3

**Fig. 3:**
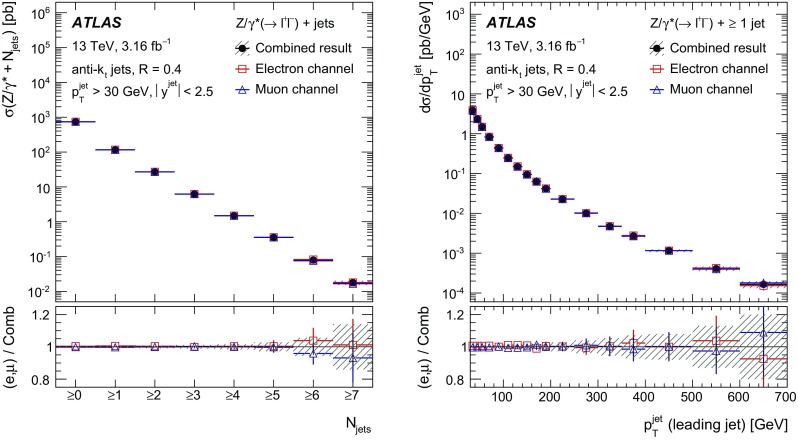
Measured fiducial cross section as a function of the inclusive jet multiplicity (*left*) and the leading jet $p_{\text {T}}$ for inclusive $Z+ \ge 1\text {~jet}$ events (*right*) in the electron and the muon channels and compared to their combined value. The ratios of the two measurements to the combined results are also shown in the *bottom panels*. The *error bars* indicate the statistical uncertainty, and the *hatched bands* the statistical and the flavour-uncorrelated systematic uncertainties of the combined result, added in quadrature

Figure 3 shows a comparison of the electron and muon channels for the measured fiducial cross section as a function of the inclusive jet multiplicity and of the leading jet $p_{\text {T}}$ for inclusive $Z+ \ge 1\text {~jet}$ events. This figure demonstrates that the results in the electron and muon channels are compatible and hence can be combined to improve the precision of the measurement. This figure also shows the result of this combination described below.

The results from the electron and muon channels are combined at dressed level for each distribution separately: inclusive and exclusive jet multiplicities, ratio for successive inclusive jet multiplicities, leading jet $p_{\text {T}}$ for $Z+ \ge 1,2,3,4\text {~jet}$ events and jet $p_{\text {T}}$ for exclusive $Z+1\text {~jet}$ events, leading jet rapidity for inclusive $Z+ \ge 1\text {~jet}$ events, $H_{\text {T}}$, $\Delta \phi _{\text {jj}}$, and $m_{\text {jj}}$. A $\chi ^2$ function whose sum runs over all measurement sets (electrons and muons), measurement points, and some of the uncertainty sources, is used for the combination [79, 80] and distinguishes between bin-to-bin correlated and uncorrelated sources of uncertainties, the latter comprising the statistical uncertainty of the data and the statistical unfolding uncertainty. Uncertainties specific to the lepton flavour and to the background are included in the $\chi ^2$ function, while the remaining, flavour-uncorrelated, systematic uncertainties related to jets, pile-up, luminosity, and unfolding are averaged after the combination.

**Fig. 4:**
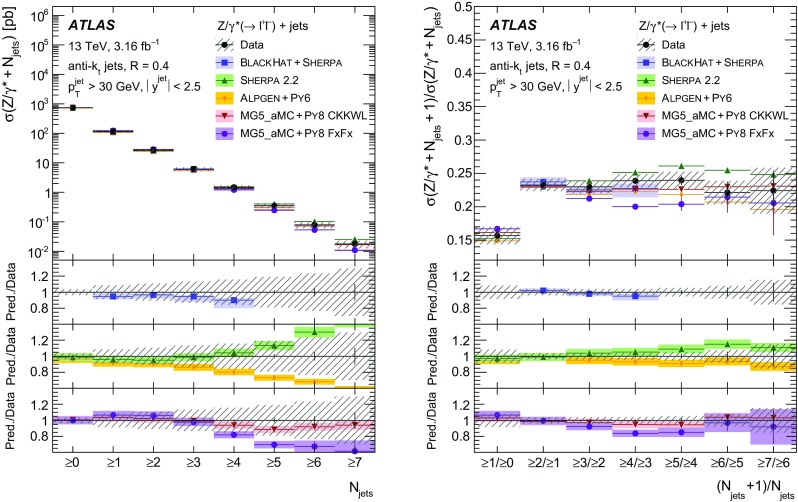
Measured cross section as a function of the inclusive jet multiplicity (*left*) and ratio for successive inclusive jet multiplicities (*right*) for inclusive $Z+\text {jets}$ events. The data are compared to the predictions from BlackHat+Sherpa, Sherpa 2.2, Alpgen+Py6, MG5_aMC+Py8 CKKWL, and MG5_aMC+Py8 FxFx. The *error bars* correspond to the statistical uncertainty, and the hatched bands to the data statistical and systematic uncertainties (including luminosity) added in quadrature. A constant 5% theoretical uncertainty is used for Sherpa 2.2, Alpgen+Py6, MG5_aMC+Py8 CKKWL, and MG5_aMC+Py8 FxFx. Uncertainties from the parton distribution functions and QCD scale variations are included in the BlackHat+Sherpa predictions, as described in Sect. 3.3

### Comparisons of results to predictions

The cross-section measurement for different inclusive $Z+\text {jets}$ multiplicities and their ratios obtained from the combination are found in Tables 5 and 6. Figure 4 shows the comparison of these results with the NLO QCD fixed-order calculations from BlackHat+Sherpa and with the predictions from Sherpa 2.2, Alpgen+Py6, MG5_aMC+Py8 CKKWL, and MG5_aMC+Py8 FxFx. The plots show the particle-level cross section with the generator predictions normalised to the inclusive NNLO cross sections in the top panel, accompanied by the ratios of the various predictions with respect to the data in the bottom panels. Uncertainties from the parton distributions functions and QCD scale variations are included in the BlackHat+Sherpa predictions, as described in Sect. 3.3. A constant 5% theoretical uncertainty is used for Sherpa 2.2, Alpgen+Py6, MG5_aMC+Py8 CKKWL, and MG5_aMC+Py8 FxFx, as described in Table 1. The inclusive jet multiplicity decreases logarithmically while the ratio is flat in the presence of at least one jet. The predictions are in agreement with the observed cross sections and their ratios, except for Sherpa 2.2, Alpgen+Py6 and MG5_aMC+Py8 FxFx for high jet multiplicity, where a non-negligible fraction of the jets are produced by the parton shower.

**Table 5:** Measured combined fiducial cross sections for successive inclusive jet multiplicities. The statistical, systematic, and luminosity uncertainties are given

Jet multiplicity	Measured cross section ± (stat.) ± (syst.) ± (lumi.) [pb]			
	$$Z \rightarrow \ell \ell $$			
${\ge }$0 jets	740 ±	1 ±	23 ±	16
${\ge }$1 jets	116.0 ±	0.3 ±	9.7 ±	2.5
${\ge }$2 jets	27.0 ±	0.1 ±	2.8 ±	0.6
${\ge }$3 jets	6.20 ±	0.04 ±	0.82 ±	0.14
${\ge }$4 jets	1.48 ±	0.02 ±	0.23 ±	0.04
${\ge }$5 jets	0.36 ±	0.01 ±	0.07 ±	0.01
${\ge }$6 jets	0.079 ±	0.004 ±	0.018 ±	0.002
${\ge }$7 jets	0.0178 ±	0.0019 ±	0.0049 ±	0.0005

**Table 6:** Measured combined ratios of the fiducial cross sections for successive inclusive jet multiplicities. The statistical, systematic, and luminosity uncertainties are given

Jet multiplicity	Measured cross-section ratio ± (stat.) ± (syst.) ± (lumi.)
	$$Z \rightarrow \ell \ell $$
${\ge }$1 jets/${\ge }$0 jets	0.1568 ± 0.0004 ± 0.0131 ± 0.0001
${\ge }$2 jets/${\ge }$1 jets	0.2327 ± 0.0011 ± 0.0093 ± 0.0002
${\ge }$3 jets/${\ge }$2 jets	0.2299 ± 0.0018 ± 0.0095 ± 0.0002
${\ge }$4 jets/${\ge }$3 jets	0.2390 ± 0.0035 ± 0.0094 ± 0.0002
${\ge }$5 jets/${\ge }$4 jets	0.2397 ± 0.0068 ± 0.0111 ± 0.0002
${\ge }$6 jets/${\ge }$5 jets	0.2213 ± 0.0127 ± 0.0123 ± 0.0003
${\ge }$7 jets/${\ge }$6 jets	0.2240 ± 0.0264 ± 0.0222 ± 0.0003

**Fig. 5:**
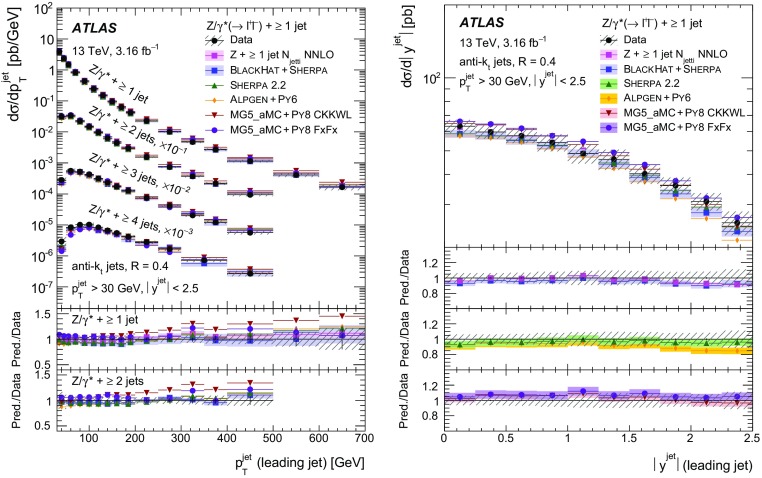
Measured cross section as a function of the leading jet $p_{\text {T}}$ for inclusive $Z+ \ge 1,2,3,4\text {~jet}$ events (*left*) and absolute value of the leading jet rapidity for inclusive $Z+ \ge 1\text {~jet}$ events (*right*). The data are compared to the predictions from $Z+ \ge 1\text {~jet~N}_{\text {jetti}}$ NNLO, BlackHat+Sherpa, Sherpa 2.2, Alpgen+Py6, MG5_aMC+Py8 CKKWL, and MG5_aMC+Py8 FxFx. The *error bars* correspond to the statistical uncertainty, and the *hatched bands* to the data statistical and systematic uncertainties (including luminosity) added in quadrature. The details of the prediction uncertainties are given in the text. For clarity, *uncertainty bands* are not shown for the Monte Carlo predictions in the *left-hand plot*. Uncertainties from the QCD scale variations for the $Z+ \ge 1\text {~jet~N}_{\text {jetti}}$ NNLO predictions are included, as described in Sect. 3.3

**Fig. 6:**
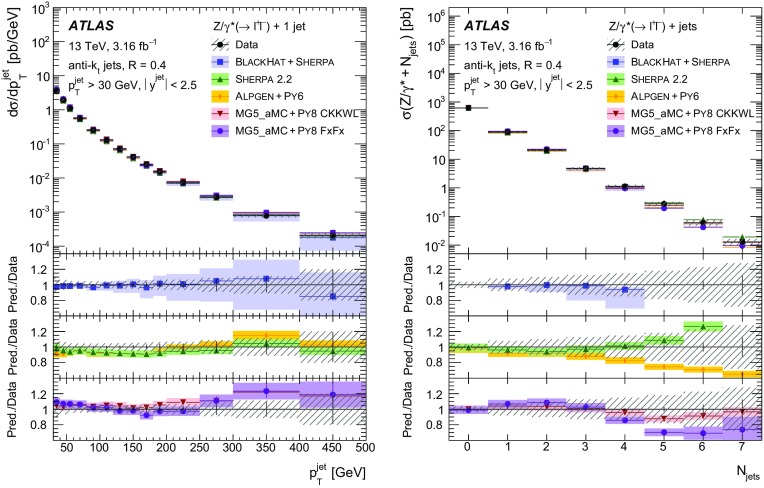
Measured cross section as a function of jet $p_{\text {T}}$ for exclusive $Z+1\text {~jet}$ events (*left*) and exclusive jet multiplicity (*right*). The data are compared to the predictions from BlackHat+Sherpa, Sherpa 2.2, Alpgen+Py6, MG5_aMC+Py8 CKKWL, and MG5_aMC+Py8 FxFx. The *error bars* correspond to the statistical uncertainty, and the *hatched bands* to the data statistical and systematic uncertainties (including luminosity) added in quadrature. The details of the prediction uncertainties are given in the text

The jet transverse momentum is a fundamental observable of the $Z+\text {jets}$ process and probes pQCD over a wide range of scales. Moreover, understanding the kinematics of jets in events with vector bosons associated with several jets is essential for the modelling of backgrounds for other SM processes and searches beyond the SM. The leading jet $p_{\text {T}}$ distribution (which is correlated with the $p_{\text {T}}$ of the $Z$ boson) in inclusive $Z+ \ge 1,2,3,4\text {~jet}$ events is shown in Fig. 5 and ranges up to 700 GeV. The LO generator MG5_aMC+Py8 CKKWL models a too-hard jet $p_{\text {T}}$ spectrum. This feature is known from studies of LO generators in *pp* collisions at lower centre-of-mass energies [11], and can be interpreted as an indication that the dynamic factorisation and renormalisation scale used in the generation is not appropriate for the full jet $p_{\text {T}}$ range. In contrast, the predictions from BlackHat+Sherpa, Sherpa 2.2, and MG5_aMC+Py8 FxFx, which are based on NLO matrix elements, are in agreement with the measured cross section within the systematic uncertainties over the full leading jet $p_{\text {T}}$ range. Alpgen+Py6 also shows good agreement with the measured data. The $Z+ \ge 1\text {~jet~N}_{\text {jetti}}$ NNLO prediction models the spectrum for the $Z+ \ge 1\text {~jet}$ events well. Uncertainties from the QCD scale variations for the $Z+ \ge 1\text {~jet~N}_{\text {jetti}}$ NNLO predictions are included in the uncertainty band, as described in Sect. 3.3. For the leading jet rapidity distribution in inclusive $Z+ \ge 1\text {~jet}$ events, also shown in this figure, all predictions show good agreement with the measured data within the uncertainties.

The exclusive jet $p_{\text {T}}$ distribution probes the validity of $Z+1\text {~jet}$ predictions at increasing QCD scales represented by the jet $p_{\text {T}}$ in the presence of a jet veto at a constant low scale; for a jet $p_{\text {T}}$ range of several hundred GeV, accessible with the current data set, the jet scale is of order ten times larger than the veto scale (30 GeV). Figure 6 demonstrates that all predictions studied are consistent with the data within systematic uncertainties over the full jet $p_{\text {T}}$ range (up to 500 GeV). This figure also shows the measured cross section as a function of the exclusive jet multiplicity, which decreases logarithmically. Similar trends as for the inclusive jet multiplicity (Fig. 4) are observed.

**Fig. 7:**
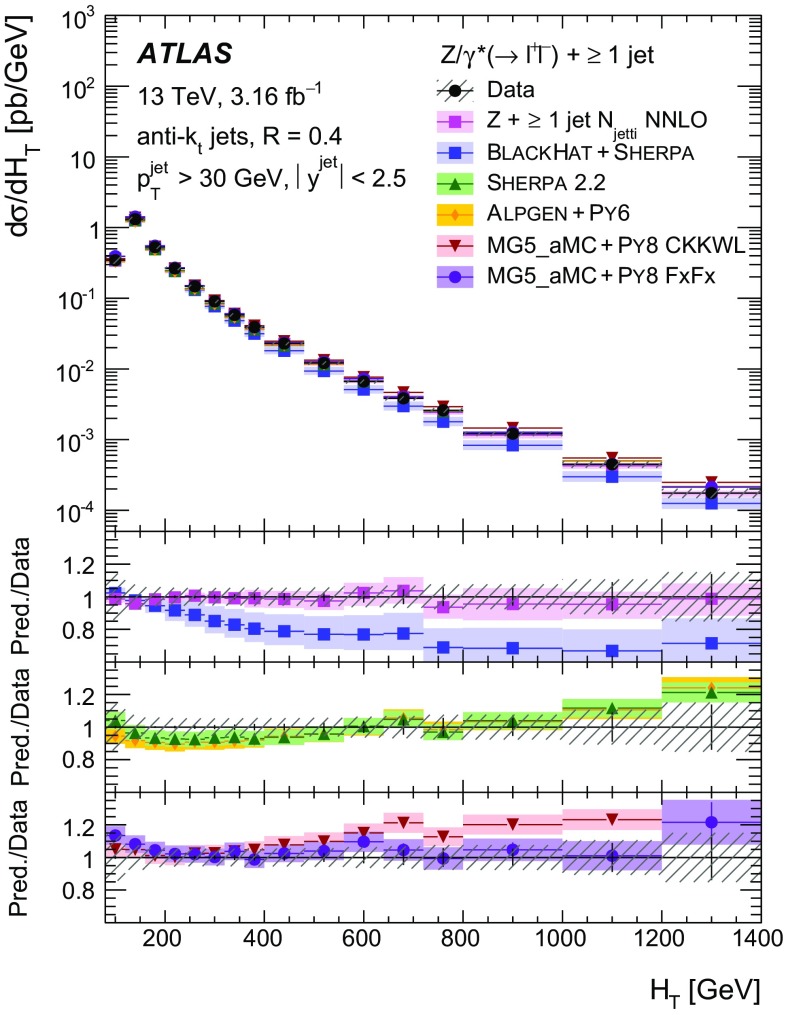
Measured cross section as a function of $H_{\text {T}}$ for inclusive $Z+ \ge 1\text {~jet}$ events. The data are compared to the predictions from $Z+ \ge 1\text {~jet~N}_{\text {jetti}}$ NNLO, BlackHat+Sherpa, Sherpa 2.2, Alpgen+Py6, MG5_aMC+Py8 CKKWL, and MG5_aMC+Py8 FxFx. The *error bars* correspond to the statistical uncertainty, and the hatched bands to the data statistical and systematic uncertainties (including luminosity) added in quadrature. The details of the prediction uncertainties are given in the text

Quantities based on inclusive $p_{\text {T}}$ sums of final-state objects, such as $H_{\text {T}}$, the scalar $p_{\text {T}}$ sum of all visible objects in the final state, are often employed in searches for physics beyond the Standard Model, to enrich final states resulting from the decay of heavy particles. The values $H_{\text {T}}$ or $H_{\text {T}}$/2 are also commonly used choices for scales for higher-order perturbative QCD calculations. Large values for this quantity can result either from a small number of very energetic particles or from a large number of less energetic particles. Figure 7 shows the measured cross sections as a function of the $H_{\text {T}}$ distribution (up to 1400 GeV) in inclusive $Z+ \ge 1\text {~jet}$ events. The predictions from Sherpa 2.2, Alpgen+Py6 and MG5_aMC+Py8 FxFx describe well the $H_{\text {T}}$ distribution. The prediction from MG5_aMC+Py8 CKKWL describes well the turn-over in the softer part of the $H_{\text {T}}$ spectrum, but overestimates the contribution at large values of $H_{\text {T}}$, in line with the overestimate of the cross sections for hard jets. The fixed-order $Z+ \ge 1\text {~jet}$ prediction from BlackHat+Sherpa underestimates the cross section for values of $H_{\text {T}} >300$ GeV, as observed in similar measurements at lower centre-of-mass energies [11, 81], due to the missing contributions from events with higher parton multiplicities, which for large values of $H_{\text {T}}$ constitute a substantial portion of the data. Agreement is recovered by adding higher orders in perturbative QCD, as demonstrated by the good description of $H_{\text {T}}$ by $Z+ \ge 1\text {~jet~N}_{\text {jetti}}$ NNLO.

**Fig. 8:**
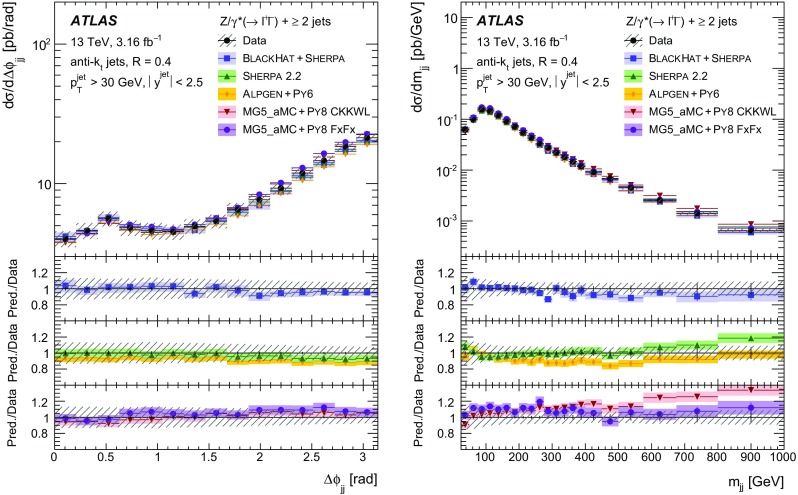
Measured cross section as a function of $\Delta \phi _{\text {jj}}$ (*left*) and $m_{\text {jj}}$ (*right*) for inclusive $Z+ \ge 2\text {~jet}$ events. The data are compared to the predictions from BlackHat+Sherpa, Sherpa 2.2, Alpgen+Py6, MG5_aMC+Py8 CKKWL, and MG5_aMC+Py8 FxFx. The *error bars* correspond to the statistical uncertainty, and the *hatched bands* to the data statistical and systematic uncertainties (including luminosity) added in quadrature. The details of the prediction uncertainties are given in the text

Angular relations between the two leading jets and the dijet mass are frequently used to separate either heavier SM particles or beyond-SM physics from the $Z+\text {jets}$ process. Figure 8 shows the differential cross section as a function of azimuthal angular difference between the two leading jets for $Z+ \ge 2\text {~jet}$ events, $\Delta \phi _{\text {jj}}$. The tendency of the two jets to be back-to-back in the transverse plane is well modelled by all predictions. This figure also shows the measured cross sections as a function of the invariant mass $m_{\text {jj}}$ of the two leading jets for $Z+ \ge 2\text {~jet}$ events. The shape of the dijet mass is modelled well by BlackHat+Sherpa, Sherpa 2.2, Alpgen+Py6, and MG5_aMC+Py8 FxFx, whereas MG5_aMC+Py8 CKKWL shows a harder spectrum.

## Conclusion

Proton–proton collision data at $\sqrt{s}=13$ TeV from the LHC, corresponding to a total integrated luminosity of 3.16 fb$^{-1}$, have been analysed by the ATLAS collaboration to study events with $Z$ bosons decaying to electron or muon pairs, produced in association with one or more jets. The fiducial production cross sections for $Z+ \ge 0$–7 jets have been measured, within the acceptance region defined by $p_{\text {T}} ^{\ell }>25$ GeV, $|\eta ^{\ell }|<2.5$, $71<m_{\ell \ell }<111$ GeV, $p_{\text {T}}^{\text {jet}} > 30$ GeV, $|y^{\text {jet}} |<2.5$, and $\Delta R(\ell ,\text {jet}) > 0.4$, with a precision ranging from 4 to 30%. Ratios of cross sections for successive jet multiplicities and cross-section measurements as a function of different key variables such as the jet multiplicities, jet $p_{\text {T}}$ for exclusive $Z+1$ jet events, leading jet $p_{\text {T}}$ for $Z+ \ge 1,2,3,4\text {~jet}$ events, leading jet rapidity for $Z+ \ge 1\text {~jet}$ events, $H_{\text {T}}$, $\Delta \phi _{\text {jj}}$ and $m_{\text {jj}}$ have also been derived.

The measurements have been compared to fixed-order calculations at NLO from BlackHat+Sherpa and at NNLO from the $Z+ \ge 1\text {~jet~N}_{\text {jetti}}$ NNLO calculation, and to predictions from the generators Sherpa 2.2, Alpgen+Py6, MG5_aMC+Py8 CKKWL, and MG5_aMC+Py8 FxFx. In general, the predictions are in good agreement with the observed cross sections and cross-section ratios within the uncertainties. Distributions which are dominated by a single jet multiplicity are modelled well by fixed-order NLO calculations, even in the presence of a jet veto at a low scale. The ME+PS generator MG5_aMC+Py8 CKKWL, which is based on LO matrix elements, models a too-hard jet spectrum, as observed in $\sqrt{s}=7$ TeV *pp* collisions. It however models well the inclusive jet multiplicity distribution over the full multiplicity range. The modelling of the jet $p_{\text {T}}$ and related observables is significantly improved by the ME+PS@NLO generators Sherpa 2.2 and MG5_aMC+Py8 FxFx, which use NLO matrix elements for up to two additional partons. The recent $Z+ \ge 1\text {~jet~N}_{\text {jetti}}$ NNLO predictions describe well key distributions such as the leading jet $p_{\text {T}}$ and $H_{\text {T}}$. The results presented in this paper provide essential input for the further optimisation of the Monte Carlo generators of Z+jets production and constitute a powerful test of perturbative QCD for processes with a higher number of partons in the final state.

## Footnotes

1 Throughout this paper, $Z /\gamma ^*$-boson production is denoted simply by $Z$-boson production.

2 ATLAS uses a right-handed coordinate system with its origin at the nominal interaction point (IP) in the centre of the detector and the *z*-axis along the beam pipe. The *x*-axis points from the IP to the centre of the LHC ring, and the *y*-axis points upwards. Cylindrical coordinates $(r,\phi )$ are used in the transverse plane, $\phi$ being the azimuthal angle around the *z*-axis. The pseudorapidity is defined in terms of the polar angle $\theta$ as $\eta = -\ln \tan (\theta /2)$. Angular distance is measured in units of $\Delta R \equiv \sqrt{(\Delta \eta )^{2} + (\Delta \phi )^{2}}$. When dealing with massive jets and particles, the rapidity $y = \frac{1}{2} \ln \left( \frac{E+p_z}{E-p_z} \right)$ is used, where *E* is the jet/particle energy and $p_z$ is the *z*-component of the jet/particle momentum.
